# Medical Doctors’ Offline Computer-Assisted Digital Education: Systematic Review by the Digital Health Education Collaboration

**DOI:** 10.2196/12998

**Published:** 2019-03-01

**Authors:** Hayfaa Abdelmageed Wahabi, Samia Ahmed Esmaeil, Khawater Hassan Bahkali, Maher Abdelraheim Titi, Yasser Sami Amer, Amel Ahmed Fayed, Amr Jamal, Nasriah Zakaria, Amna Rehana Siddiqui, Monika Semwal, Lorainne Tudor Car, Paul Posadzki, Josip Car

**Affiliations:** 1 Research Chair of Evidence-Based Healthcare and Knowledge Translation Deanship of Research King Saud University Riyadh Saudi Arabia; 2 Department of Family and Community Medicine College of Medicine King Saud University Riyadh Saudi Arabia; 3 Patient Safety Unit, Quality Management Department King Khalid University Hospital King Saud Medical City Riyadh Saudi Arabia; 4 Clinical Practice Guidelines Unit, Quality Management Department King Khalid University Hospital King Saud University Medical City Riyadh Saudi Arabia; 5 College of Medicine Princess Nourah Bint Abdulrahman University Riyadh Saudi Arabia; 6 High Institute of Public Health Alexandria University Alexandria Egypt; 7 Medical Informatics and e-Learning Unit, Medical Education Department College of Medicine King Saud University Riyadh Saudi Arabia; 8 Department of Community Health Sciences Aga Khan University Karachi Pakistan; 9 Centre for Population Health Sciences Lee Kong Chian School of Medicine Nanyang Technological University Singapore Singapore; 10 Family Medicine and Primary Care Lee Kong Chian School of Medicine Nanyang Technological University Singapore Singapore

**Keywords:** systematic review, medical education, digital education

## Abstract

**Background:**

The widening gap between innovations in the medical field and the dissemination of such information to doctors may affect the quality of care. Offline computer-based digital education (OCDE) may be a potential solution to overcoming the geographical, financial, and temporal obstacles faced by doctors.

**Objective:**

The objectives of this systematic review were to evaluate the effectiveness of OCDE compared with face-to-face learning, no intervention, or other types of digital learning for improving medical doctors’ knowledge, cognitive skills, and patient-related outcomes. Secondary objectives were to assess the cost-effectiveness (CE) of OCDE and any adverse effects.

**Methods:**

We searched major bibliographic databases from 1990 to August 2017 to identify relevant articles and followed the Cochrane methodology for systematic reviews of intervention.

**Results:**

Overall, 27 randomized controlled trials (RCTs), 1 cluster RCT (cRCT), and 1 quasi-RCT were included in this review. The total number of participants was 1690 in addition to the cRCT, which included 24 practices. Due to the heterogeneity of the participants, interventions, and outcomes, meta-analysis was not feasible, and the results were presented as narrative summary.

Compared with face-to-face learning, the effect of OCDE on knowledge gain is uncertain (ratio of the means [RM] range 0.95-1.17; 8 studies, 495 participants; very low grade of evidence). From the same comparison, the effect of OCDE on cognitive skill gain is uncertain (RM range 0.1-0.9; 8 studies, 375 participants; very low grade of evidence). OCDE may have little or no effect on patients’ outcome compared with face-to-face education (2 studies, 62 participants; low grade of evidence).

Compared with no intervention, OCDE may improve knowledge gain (RM range 1.36-0.98; 4 studies, 401 participants; low grade of evidence). From the same comparison, the effect of OCDE on cognitive skill gain is uncertain (RM range 1.1-1.15; 4 trials, 495 participants; very low grade of evidence). One cRCT, involving 24 practices, investigated patients’ outcome in this comparison and showed no difference between the 2 groups with low-grade evidence.

Compared with text-based learning, the effect of OCDE on cognitive skills gain is uncertain (RM range 0.91-1.46; 3 trials with 4 interventions; 68 participants; very low-grade evidence). No study in this comparison investigated knowledge gain or patients’ outcomes.

One study assessed the CE and showed that OCDE was cost-effective when compared with face-to-face learning if the cost is less than or equal to Can $200. No trial evaluated the adverse effect of OCDE.

**Conclusions:**

The effect of OCDE compared with other methods of education on medical doctors’ knowledge and cognitive skill gain is uncertain. OCDE may improve doctors’ knowledge compared with no intervention but its effect on doctors’ cognitive skills is uncertain. OCDE may have little or no effect in improving patients’ outcome.

## Introduction

### Background

Faced with the rapid innovations in medicine, structured postgraduate residency programs, in addition to continuing professional development (CPD) and continuing medical education, (CME), have been developed to advance and update the skills and knowledge of medical doctors and other health professions [1]. Evidence suggested that these programs are effective in improving the diagnostic and therapeutic competencies of health care professionals and patient-related outcomes [2-6]. Although face-to-face learning is the dominant method of teaching in these programs, it is being increasingly supplemented or replaced with digital learning using both Web-based and offline options [7]. Digital education involves the delivery of educational material through Information and Communication Technology using a wide variety of pedagogical designs and formats [8,9]. Digital learning is a plausible low-cost platform that provides convenient access to educational materials with flexibility in terms of pace, place, and time [10]. It has many advantages over traditional learning, such as outreach, flexibility, and adaptability, as it has the potential to reach a large number of learners regardless of the physical distance; in addition, it caters for the pace and time of the individual participant while reducing the overhead costs of the learning process [11-13]. These features of digital learning could prove it to be a plausible solution to the constraints faced by medical education in low- and middle-income countries (LMICs) where digital learning has been used effectively to improve existing health services [12] and the diagnostic and therapeutic competencies of the health care providers [2].

Despite the potential of digital learning to be the leading learning method in sciences, including medical education, some disadvantages have been reported for this method of learning. Lack of interaction with other learners and the instructor, because of flexibility in the timing of learning, may lead to social isolation and discouragement of team work [11,14].

Although digital learning caters for students’ learning pace, it tends to deindividualize the instructor and fails to respond to the learners’ individual needs [15]. Although digital learning is associated with reduced cost for the learner, the potentially large cost of designing some courses, such as those that involve virtual reality and simulation, cannot be ignored [15].

Unlike other learning methods, digitally-based courses must be designed carefully to meet the instructional objectives as the absence of an instructor for explanation makes such courses more susceptible to design flaws [15,16]. For effective digital learning in medical education, many essential design characteristics have been suggested, such as effective communication among the learners and validation and assessment of knowledge gained in addition to use of real-world scenarios [17].

Currently, there are many different technological platforms for digital learning, including Web-based learning activities, computer-based instruction, and mobile learning. The availability of many platforms paved the way for many learning opportunities in medical education, such as massive open Web-based courses, serious gaming and gamification, and virtual patient-based learning. The focus of this review is offline computer-based digital education (OCDE).

This type of digital education does not require internet or local area network connection, and the learning material is typically kept in either magnetic storage such as floppy discs or optical storage such as CD, digital versatile disc, flash memory, multimedia cards, and external hard discs, which facilitates the delivery of various educational material such as text, images, audio, and video material [18-20]. OCDE has many advantages, especially in settings where internet access is absent or limited. It has many of the advantages over other digital learning platforms as it provides a solution for those learners faced with geographical, financial, and temporal barriers to face-to-face education without compromising on the teaching and learning process or the intended outcomes [18,19,21].

### Objectives

The objective of this systematic review was to evaluate the effectiveness of OCDE compared with face-to-face learning, no intervention, text-based learning, or other type of digital learning for improving medical doctors’ knowledge, cognitive skills, and patient-related outcomes. The secondary objectives were to assess the cost and cost-effectiveness (CE) of OCDE and adverse effects of the interventions.

## Methods

### Eligibility Criteria

#### Types of Studies

We included randomized controlled trials (RCTs), cluster RCTs (cRCTs), and quasi-randomized trials that compared OCDE (personal computer or laptop) methods for medical doctors and dentists with face-to-face learning, written information, no intervention, or other OCDE. We excluded crossover trials because of the high likelihood of a carry-over effect [22]. We included trials reported in conference proceedings and abstracts when information could be obtained by contacting the authors. For a detailed description of the methodology, please refer to the study by Car et al [23].

#### Types of Participants

We included studies in which participants (learners) were medical doctors and dentists who were enrolled in postgraduate medical education programs. Studies with mixed participant groups, such as doctors and nurses, in which results for medical doctors could not be obtained separately, were excluded.

#### Types of Interventions

We included studies in which OCDE interventions were used to deliver educational content. Residency training programs and CME- and CPD-based programs that involved the use of OCDE interventions were included [24,25].

OCDE intervention refers to the use of personal computers or laptops that have assisted in the delivery of standalone multimedia materials without the need for internet or local area network connections [24,25].

We only considered studies that made the following intervention comparisons:

- Offline-based intervention versus traditional face-to-face learning.
- Offline-based intervention versus no intervention.
- Offline-based digital learning versus written text-based learning.
- Offline-based digital learning versus another method of digital learning.

#### Primary Outcomes

We included studies that reported at least one of the following primary or secondary outcomes

- Learner’s knowledge: defined as the learners’ factual or conceptual understanding.
- Learner’s cognitive skills: defined as skills used in the process of acquiring knowledge, for example, skills learned for reading an x-ray film or learning the steps of performing a procedure.
- Patients’ outcomes: defined as the direct observation of the application of knowledge and skills on the patients where the outcome of the trial is patients’ physical, mental, and psychological condition, such as the clinical effect of optimizing medication regimens. In addition, we considered studies that aimed at improving health services, such as improving existing screening programs, as patients’ outcomes.

#### Secondary Outcomes

- CE of digital learning
- Any adverse outcome

#### Types of Outcome Measures

These outcomes were assessed using any validated or non-validated instrument to measure the difference in pre and posttest scores. These assessments were either subjective (eg, self-reported) or objective (eg, questionnaire). When several posttest results were available, data were recorded as to when those tests were conducted and the difference between the pretest and the first posttest was used for the analysis.

### Search Methods

We searched major bibliographic databases from 1990, when the virtual learning environments began and schools started delivering Web-based courses, till August 2017 to identify all relevant articles. We searched in English but included papers published in any language.

#### Electronic Searches

We searched the following databases: Medical Literature Analysis and Retrieval System Online (MEDLINE, via Ovid), EMBASE (via Ovid), Web of Science, Educational Resource Information Centre (via Ovid), Cochrane Central Register of Controlled Trials, Cochrane Library, PsycINFO (Ovid), Cumulative Index to Nursing and Allied Health Literature (via EBSCO), and ProQuest Dissertation and Theses Database. The MEDLINE search strategy was adapted to search other databases (Multimedia Appendix 1).

#### Searching Other Resources

We searched the reference lists of all included studies and relevant systematic reviews. We also searched the International Clinical Trials Registry Platform Search Portal and Current Controlled Trials metaRegister of Controlled Trials to identify the unpublished trials and contacted relevant investigators for further information.

### Data Collection and Analysis

#### Study Selection

Two reviewers independently screened the titles and abstracts and identified studies potentially meeting the inclusion criteria. The full-text versions were retrieved and read in full. Finally, 2 review authors independently assessed the articles against the eligibility criteria. Any disagreements were resolved through discussion between the 2 authors. If no agreement was reached, a third author acted as an arbiter. Two reviewers verified the final list of included studies.

#### Data Extraction and Management

Two reviewers independently extracted and managed the data for each of the included studies and used a structured data recording form. In addition to the usual information on the study design and participants’ demographics, we extracted data on relevant fields such as the country where the trial was conducted, funding source, and duration of intervention. Disagreements between the review authors were resolved by discussion. A third review author acted as an arbiter in case disagreements were not resolved.

#### Dealing with Missing Data

Whenever possible, we attempted to obtain missing data from the original authors.

#### Assessment of Risk of Bias in Included Studies

Two reviewers independently assessed the risk of bias of each of the included studies using the Cochrane Collaboration’s risk of bias tool [22]. Studies were assessed for the risk of bias in the following domains: random sequence generation, allocation concealment, blinding of participants or personnel, blinding of outcome assessors, completeness of outcome data (attrition bias), selective outcome reporting (reporting bias), and other sources of bias including baseline imbalance and contamination. For cRCTs, we assessed and reported the risk of bias associated with an additional domain: selective recruitment of cluster participants, baseline imbalance, attrition of clusters, and not accounting for cluster effect in analysis [26].

We judged the risk of bias for each study to be of 1 of 3 levels: high, low, or unclear risk of bias. We scored each study for risk of bias as follows: *low* if all key domains were scored as *low risk* or if 1 domain is scored as *unclear*. We scored the trial as *unclear* if 2 key domains were scored as *unclear* *risk* and *high* if more than 2 key domains were scored *unclear risk* or 1 domain scored *high* *risk*, adapted from the study by Davey et al [27].

Reporting bias was assessed qualitatively on the basis of the characteristics of included studies. Due to the heterogeneity of the trials (in terms of populations, interventions, comparator groups, and outcomes), data pooling was not feasible.

We used Evers checklist [28] to evaluate risk of bias in articles that examined the CE of OCDE.

#### Data Synthesis

We reported post intervention values for the outcomes of intervention and control groups and the effect size as reported by the authors (*P* value). In addition, we calculated the ratio of the means (RM) [4] whenever feasible. As the heterogeneity of populations, outcomes, and comparisons precluded meta-analysis, we provided a narrative summary of the results.

#### Summary of Findings Tables

For main comparisons, 2 authors used the *Grading of Recommendations Assessment*,  *Development and Evaluation* (GRADE) criteria independently of one another to assess the quality of evidence [29]. We considered the following limitations: risk of bias, inconsistency of results, indirectness of the evidence, imprecision, or publication bias, and subsequently downgraded the quality of evidence where appropriate [30].

## Results

### Overview

**Figure 1:**
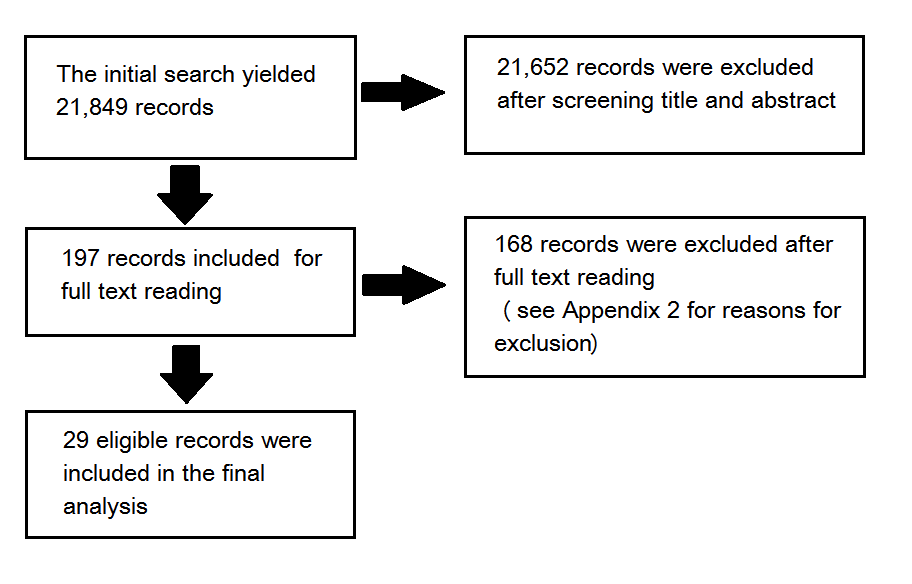
Preferred Reporting Items for Systematic Reviews and Meta-Analyses (PRISMA) flow diagram of the trial selection.

The study selection process is shown in the Preferred Reporting Items for Systematic Reviews and Meta-Analyses (PRISMA) flow diagram (Figure 1).

The initial search yielded 21,849 records. After screening the titles and abstracts, we obtained the full-text reports for 197 records and assessed them for inclusion in the review. Of these, we excluded 168 studies that did not meet the eligibility criteria (Multimedia Appendix 2). The remaining 29 articles were included in this review.

We contacted authors of abstracts for further information and data on their trials [31-37], we received a response from 2 authors, Rae et al [37], where the data were subsequently included in the review, and Ukabiala et al [36], which was subsequently excluded. We tried to contact the authors of the cRCT [38] for data and information to assess the risk of bias and to calculate the intracluster correlation coefficients (ICC); however, the contact information was void.

### Included Studies

A total of 24 of the 29 included trials were parallel RCTs, each included 2 arms and 1 trial had 4 arms, 3 of which were included in this review, resulting in 2 interventions [37]. In addition, 1 trial had 3 arms [39], 1 was a factorial RCT [40], 1 was a cRCT [38], and 1 was a quasi-RCT [41]. All trials were published in peer-reviewed journals except for 1 trial where unpublished data were obtained from the authors [37]. Overall, 24 studies (83%, 24/29) were conducted in high-income countries [37-60], and the remaining 5 studies (17%, 5/29) were conducted in upper middle-income countries [61-65].

Two studies investigated OCDE in dentistry [39,45], whereas the rest investigated it in medicine, including 6 in surgery or anesthesia [37,47,48,50,51,54], 7 in internal medicine or family medicine [40,44,49,53,57,60,65], 5 in pediatrics [38,41,46,56,58], 3 in psychiatry [52,61,63], 1 in obstetrics and gynecology [42], and 1 in radiology [43]. In the 4 remaining trials, the subject of intervention was evidence-based medicine [55] or advanced life support [59,62,64]. We included 1 cRCT in this review [38]. We obtained data at the participants’ level to estimate the outcome effect; however, the effect of clustering was not adjusted for and sufficient information was not available to perform reanalysis to account for ICC (Multimedia Appendix 3).

### Participants’ Characteristics

The total number of participants included across all trials was 1690 in addition to 1 cRCT [38], which was conducted in 24 medical practices, but the number of participants was not specified. The study with the largest number of participants included 88 dentists in the control group and 86 in the intervention group [45], and the smallest study included 5 participants in the control and 6 participants in the intervention group [58]. Most of the participants were trainees. In addition, 4 studies were conducted among 377 medical interns [55,62-64], 16 trials among a total of 686 postgraduate residents [37,42,44,46-50,52,54,58-61,65], and 1 study included 49 residents and faculty members [43], of the remaining studies, 7 were performed on 578 practicing doctors [39-41,45,53,56,57] (Multimedia Appendix 3).

### Intervention Characteristics

Overall, 15 studies compared OCDE with classroom or face-to-face learning [41,43,47-51,53-55,60-64], 3 studies with 4 interventions [37,46,58] compared OCDE with text-based resources, and 9 studies [38-40,42,44,45,52,56,59] compared OCDE with no intervention. The other 2 trials compared OCDE with another OCDE [57,65]. In addition, 23 trials reported the duration of the exposure to the intervention [38,40-42,46-51,53-65], which ranged between 2.5 min [58] and 12 months [38,59] (Multimedia Appendix 3).

All the studies used OCDE that was delivered by either personal computers or laptops. In addition, 19 studies used software- or computer-based programs delivered via a variety of sources such as CD-ROM and stored in the computer [38-41,44,46,47,50,52,53,55-57,59,60,62-65], 6 studies used video recording [42,48,49,51,58,61], 3 studies [43,45,54] investigated the use of multimedia, and 1 study used both computer-based program and video recording [37].

#### Primary Outcomes

As meta-analysis was not feasible, we presented the results in a narrative summary format (Multimedia Appendix 4) and Summary of Findings (Multimedia Appendix 3).

##### Doctors’ Knowledge

Among the 29 studies that compared OCDE with other interventions or to no intervention, knowledge was assessed in 13 studies (44%) [41-45,47,49,55,59,60,63-65]. Knowledge gain was assessed by multiple choice questions (MCQs) in 5 studies [42,47,59,63,64] and by test of true or false questions in 2 studies [44,60], none of the tests were validated. Either non-validated or low-internal validity open-ended questions were used in 3 studies to assess knowledge [43,49,65]: 1 study used a validated questionnaire comprising MCQs and structured questions [55], 1 used non-validated Likert scale questions [45], and 1 used invalidated anonymous scoring by the authors compared with a gold standard prepared by 3 neurologists [41] (Multimedia Appendix 3).

##### Doctors’ Cognitive Skills

Overall, 16 RCTs with 17 interventions [37,39,43,45-48, 50,52-54,56,58,61,62,65] assessed cognitive skills as an outcome. A total of 3 studies [48,50,54] used the validated anesthetist nontechnical skills scoring system to assess doctors’ cognitive skills. One study [62] used the objective structured clinical examination (OSCE) tool for evaluation of skills. Four studies [52,53,58,65] used thematic analysis to assess skills gain, including thinking process [58], task completion rate [65], number of empathetic statement responses to patients [53], and agreement between expert and participant in mental health capacity assessment [52].

Cognitive skills gain was assessed by many tools including calculating the accuracy of decision making within and between the study groups [39], questionnaires [45,56], and a 36-item checklist [37]. Multiple assessment tools were used in 2 studies to assess cognitive skills, Ottolini et al [46] used 2 tools, thematic analysis and a questionnaire, whereas Esfahani et al [61] used both the Jefferson empathy scale and OSCE. A questionnaire with open-ended questions was used in 1 study [43], and an MCQ test was used in another [47] (Multimedia Appendix 3).

##### Patients’ Outcomes

A total of 4 studies [38,40,53,57] assessed patient outcomes. Bonevski et al [57] examined doctors’ improved screening and detection of the patients’ risk behaviors using a self-reported survey. Millard et al [40] evaluated the improvement in dementia diagnosis, following a computer-generated audit of the participants’ practices. Tulsky et al [53] used a telephone survey to evaluate the patients’ trust in their oncologists and the oncologists’ perceived empathy and knowledge of the patients, following CD-ROM–based education. Lavigne et al [38] evaluated patients’ outcomes by measuring the improvement in the children’s attention-deficit/hyperactivity disorder (ADHD) symptoms using the Beck Anxiety Inventory, Swanson, Nolan, and Pehlam-IV Rating Scale, and the ADHD Rating Scales-IV completed by teachers and parents following the use of computer software education in medication dose titration (Multimedia Appendix 3).

#### Secondary Outcomes

The CE of OCDE computer-based digital learning was examined in 1 study [51]. The study used data from an RCT included in this review [48]. It compared the cost and effectiveness of self-debriefing versus instructor debriefing using net benefit regression. The CE estimate was reported as the incremental net benefit, and the uncertainty was presented using a CE acceptability curve. The study concluded that digital learning was cost-effective if the intervention cost was less than or equal to Can $200 in the 2012 rate.

We did not find any RCT that compared the adverse effects of OCDE with other interventions.

### Risk of Bias in Included Studies

**Figure 2:**
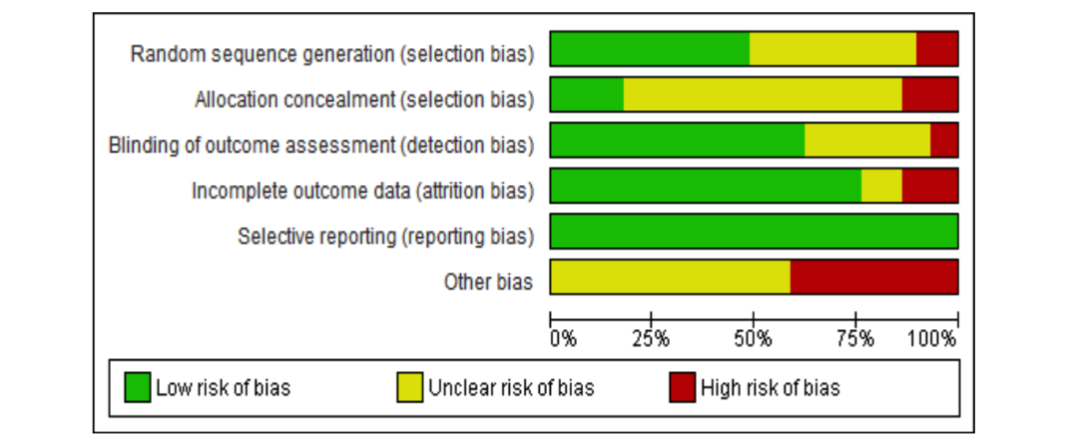
Risk of bias graph.

**Figure 3:**
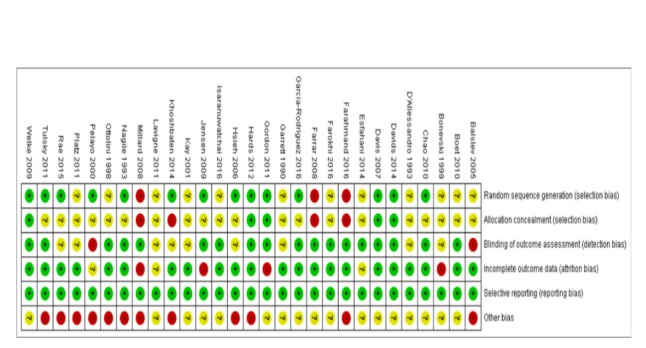
Risk of bias summary.

The assessment of risk of bias is described in detail in Multimedia Appendix 4 and shown on Figures 2 and 3. A total of 24 of the 29 included trials (83%) were judged to be at a high risk of bias [37-41,43-48,50-53,56-64]. In addition, 2 trials were judged to be of unclear risk of bias [42,49], whereas only 3 trials were judged to be at low risk of bias [54,55,65]. The assessment of the methodological quality of economic evaluation using the Evers checklist is presented in Multimedia Appendix 5.

### Effects of the Interventions

The studies were divided into 4 comparisons, which evaluate the impact of OCDE compared with face-to-face learning, with no intervention, OCDE with text-based learning, and with another OCDE method.

#### Offline Computer-Based Digital Education Compared With Face-To-Face Learning

The characteristics of the studies are presented in (Multimedia Appendix 3); GRADE of evidence is presented in Summary of Findings (Multimedia Appendix 6).

##### Knowledge Gain

Overall, 4 studies [43,49,55,64] showed no significant difference in posttest knowledge scores between digital learning and face-to-face learning (ratio of the mean [RM] ranges from 1.0 to 1.13). In addition, 3 studies [41,60,63] showed the OCDE group to have significantly higher scores than the face-to-face group. However, the difference among the posttest mean scores of the participants was modest, as indicated by the small RM (RM=1.1, RM=1.17, and RM=1.13, respectively). One study [47] showed face-to-face participants to score higher than OCDE group (RM=0.95). The grade of evidence for this outcome is very low because of the high risk of bias in the included studies, the heterogeneity of participants and interventions, and the indirectness of evidence; therefore, it is uncertain whether there is difference in knowledge gain between OCDE and face-to-face learning.

##### Cognitive Skills Gain

Overall, 4 RCTs showed no significant difference between OCDE and face-to-face learning in posttest mean scores of skills gain [43,48,50,54] (RM range 0.94-1.0). In 2 studies [47,61], the mean posttest scores of face-to-face participants were significantly higher than those of the OCDE intervention (RM 0.91 and 0.95, respectively). In addition, 2 studies [62,53] showed the mean posttest score for the participants of the OCDE to be significantly higher than that of the face-to-face controls. The grade of evidence for this outcome is very low because of the high risk of bias in the included studies, the heterogeneity of participants and interventions, and the indirectness of evidence; therefore, it is uncertain whether there is a difference in cognitive skill gain between OCDE and face-to-face learning.

##### Patients’ Outcomes

Patients’ outcomes were examined in 2 trials [40,53]. Patients’ outcomes significantly improved in the OCDE group. The grade of evidence for this outcome is low because of the high risk of bias and heterogeneity of participants and interventions; therefore, OCDE may improve patients’ outcome compared with face-to-face learning.

#### Offline Computer-Based Digital Education Compared With No Intervention

The characteristics of the studies are presented in (Multimedia Appendix 3); GRADE of evidence is presented in Summary of Findings (Multimedia Appendix 6).

##### Knowledge Gain

Four RCTs [42,44,45,59] investigated the effect of OCDE compared with no intervention on knowledge gain. Of them, 3 trials [42,44,45] showed that OCDE was significantly more effective than no intervention with modest effect (RM range 1.11-1.36). The fourth trial [59] showed no significant difference in the posttest knowledge scores between intervention and control (RM=0.98). The grade of evidence for this outcome is low because of the high risk of bias and heterogeneity of participants and interventions; therefore, digital learning may improve knowledge gain compared with no intervention.

Overall, 3 trials [39,45,52] showed that OCDE had similar effect to no intervention in cognitive skills gain (RM=1.01), whereas Gordon et al [56] showed that participants in OCDE had significantly higher posttest scores compared with control (RM=1.25). The grade of evidence for this outcome is very low because of the high risk of bias, heterogeneity of participants and interventions, and indirectness of evidence; therefore, there is uncertainty about the effectiveness of OCDE compared with no intervention in cognitive skill gain.

##### Patients’ Outcomes

Only 1 cRCT, at high risk of bias, investigated the effect of OCDE compared with no intervention on patients’ outcome [38]. The trial was conducted among doctors of 24 pediatric practices where the number of doctors was not specified. It showed similar effectiveness in the treatment of patients with ADHD in intervention and control groups. The grade of evidence for this outcome was low because of the high risk of bias and the fact that evidence was drawn from a single study; therefore, OCDE may have an equal effect as no intervention in patients’ outcomes.

#### Offline Computer-Based Digital Education Compared With Text-Based Learning: Cognitive Skills Gain

The characteristics of the studies are presented in Multimedia Appendix 3; GRADE of evidence is presented in Summary of Findings Table (Multimedia Appendix 6).

Overall, 3 RCTs with 4 interventions [37,46,58] investigated the effect of OCDE compared with text-based learning on cognitive skills gain. In the 3 comparisons [37,46,58], OCDE was significantly more effective than text (RM range 1.14-1.46). In the fourth comparison [37], there was no difference in the posttest scores between the intervention and control groups (RM=0.91). The grade of evidence is very low because of the high risk of bias, small number of participants, and indirectness of evidence; therefore, there is uncertainty about the effect of OCDE compared with text-based learning in cognitive skills gain.

#### Offline Computer-Based Digital Education Compared With Other Digital Learning

The characteristics of the studies are presented in Multimedia Appendix 3.

##### Knowledge Gain

Only 1 trial at low risk of bias investigated knowledge gain [65]. It showed no difference in the effects of 2 methods of digital learning (RM=0.98). The body of evidence is considered low grade as the evidence is driven from a single study with a small number of participants.

##### Cognitive Skills Gain

Only 1 RCT, at low risk of bias, in this comparison investigated cognitive skill gain [65]. It showed equal effects from 2 methods of OCDE (RM=0.98). The body of evidence is considered low grade as the evidence is driven from a single study with a small number of participants.

##### Patients’ Outcomes

One RCT compared offline computer-based CME with feedback with the same CME without feedback in improving screening behavior as patients’ outcome [57]. The RCT showed better patients’ outcomes for CME with audit compared with the same program without audit. The body of evidence is considered very low grade as the evidence is driven from a single study at high risk of bias with a small number of participants.

## Discussion

### Principal Findings

This systematic review showed that the effectiveness of OCDE compared with other methods of education, on medical doctors’ knowledge and cognitive skill gain, is uncertain. OCDE may improve doctors’ knowledge compared with no intervention, but its effect on doctors’ cognitive skills is uncertain. OCDE may have little or no effect in improving patients’ outcomes.

The evidence for this review is driven from 29 RCTs, which covered a wide range of offline digital learning interventions in a variety of clinical and nonclinical medical disciplines. The studies investigated multiple outcomes of the intervention in 1690 doctors and dentists; therefore, they provide a considerable body of evidence. However, heterogeneity of participants, interventions, and methods of assessment of outcomes, in addition to the poor methodological quality of the trials, resulted in uncertainty about the effectiveness of OCDE compared with other instruction methods.

The quality of evidence for all outcomes was rated as low or very low (for different outcomes) on the GRADE scale because of the poor methodological quality of the included studies, as 24 out of the 29 included studies were judged to be at high risk of bias in addition to the marked clinical heterogeneity of the body of evidence.

It is worth noting that in all trials that compared OCDE with other types of learning, the measured outcome was the participants’ improved knowledge or skills (contents) rather than the methods of learning. This surrogate outcome (indirectness of evidence) is valid for the evaluation of the methods of learning as long as the assumption that the participants in the intervention and control groups had equal baseline knowledge with respect to the contents of the interventions is valid. Nevertheless, bias can be introduced if the participants have different levels of knowledge about the contents (eg, same content was taught in medical school, participants at different level of training) and no pre-intervention test was performed or was completed with an invalidated tool, which is the case in most of the trials included in this review. Furthermore, the body of evidence in this review has been drawn from small individual studies, as 12 of the included studies had less than 50 participants.

The external validity of the interventions in this review has been compromised by the recruitment of volunteers, which might have resulted in the selection of participants who were more computer literate, and therefore overestimated the effect of offline OCDE by excluding participants who did not know how to use the technology or were unwilling to do so. We believe variation in computer literacy and cultural differences may influence the generalization of our results to LMICs. Furthermore, most of the included studies were experimental trials conducted in ideal university hospital settings rather than implemented in programs in the field; therefore, the true applicability of OCDE could not be examined by this review.

The results of this review are inconsistent with previous evidence about the effectiveness of digital learning for health care professionals in improving knowledge and skills gain. A systematic review of 15 RCTs on the effects of digital learning (both on the Web and offline) showed digital learning to outperform or have equal effects as face-to-face learning in knowledge gain and practice improvement [10]. Similar effects of Web-based continuing education compared with face-to-face learning for medical doctors were found by Wulto et al [66] in their systematic review, which included 16 RCTs. Another systematic review [67], which examined the effectiveness of computer-based programs on the dentists’ performance, time spent, and attitude toward the programs, showed that in all the 12 included studies except 1, the computer-based programs were either better or similar to face-to-face learning in knowledge gain and that dentists had a positive attitude toward the program. More recent systematic reviews that investigated the effectiveness of a specific type of digital learning on the knowledge and skills gain of health professionals have shown similar results [68,69]. However, our conclusion of the effect of OCDE has been based on grading the evidence base of the effectiveness of OCDE, which we believe gives a more accurate evaluation of the effectiveness of the intervention on the desired outcomes. To that end, our conclusion agrees with a recently published Cochrane systematic review [70], which considered grading of the body of evidence an integral part of its conclusion.

### Strengths and Limitations

To complete this review, we followed the robust methodology outlined by the Cochrane collaboration for searching, assessing, and reporting of the body of evidence for the effectiveness of OCDE in improving medical doctors’ knowledge and cognitive skills.

The review comprehensively evaluated the OCDE for medical doctors and dentists. The participants of the included studies are representative of the target population of medical doctors and dentists in training and non-training posts. Furthermore, the interventions accommodated participants from almost all clinical disciplines in addition to 4 fields of general knowledge and skills, including evidence-based medicine, research methodology, advanced life support, and cardiopulmonary resuscitation.

However, the heterogeneity of the participants, interventions, and outcomes precluded meta-analysis and subgroup analysis, which would have improved our certainty about the effectiveness of the intervention.

### Implications for Practice and Research

The uncertainty associated with the effectiveness of OCDE for medical doctors’ education calls for limited-scale implementation of OCDE in the context of experimental settings and research.

Research in digital education should be employed to investigate effectiveness in updating medical doctors’ knowledge and skills, considering the patient as the center of care and the improvement of patients’ health as the main outcome, especially in LMICs. Future trials should follow a robust methodology, focusing on avoiding major biases by employing valid methods for randomization and allocation concealment, in addition to the use of validated tests to assess the outcomes. As most of the participants are not blinded to the intervention in these types of studies, there is high risk of attrition bias for any outcome that relied on active participation and follow-up (eg, demonstrating skills or taking a knowledge test). Such bias can be reduced by securing the anonymity of the participants, for example, replacing their names with numbers or letters.

The indirectness of evidence will continue to downgrade the evidence base of the effectiveness of OCDE unless validated pre and posttests are used to evaluate the outcomes in addition to attentive selection of trial participants who have no previous knowledge about the subject of the education (eg, new imaging technique).

Furthermore, we believe that the development of a common taxonomy for digital learning will facilitate easier comparison among studies and therefore better the quality of evidence.

Evaluating the CE of the various methods of digital learning is an important field for future research, considering the need of such interventions in LMICs.

### Conclusions

The effectiveness of OCDE when compared with other methods of education, on medical doctors’ knowledge and cognitive skill gain, is uncertain. OCDE may improve doctors’ knowledge when compared with no intervention, but its effect on doctors’ cognitive skills is uncertain. OCDE may have little or no effect in improving patients’ outcome.

## Appendix

Multimedia Appendix 1 Search Strategy.

Multimedia Appendix 2 Excluded studies.

Multimedia Appendix 3 Characteristics of the included studies.

Multimedia Appendix 4 Risk of bias summary.

Multimedia Appendix 5 Evers checklist for economic evaluation.

Multimedia Appendix 6 Summary of findings tables.

## Abbreviations

ADHD: attention-deficit/hyperactivity disorder
CE: cost-effectiveness
CME: continuing medical education
CPD: continuing professional development
cRCT: cluster randomized controlled trial
GRADE: Grading of Recommendations Assessment, Development and Evaluation
LMICs: low- and middle-income countries
MCQs: multiple choice questions
MEDLINE: Medical Literature Analysis and Retrieval System Online
OCDE: offline computer-based digital education
OSCE: objective structured clinical examination
RCT: randomized controlled trial
RM: ratio of the means
ICC: intracluster correlation coefficients
