# Supplementary material for: Medical Doctors’ Offline Computer-Assisted Digital Education: Systematic Review by the Digital Health Education Collaboration
Source: J Med Internet Res. 2019 Mar 1;21(3):e12998. doi: 10.2196/12998 (PMC6418481; doi:10.2196/12998)
Supplement: Multimedia Appendix 2 [file jmir_v21i2e12998_app2.pdf]

Medical doctors' offline-computer-assessed digital education: a systematic review by the Digital Health Education collaboration

Online Supplementary Document 2

Table 1: Excluded studies and reason for exclusion:

NO	Study	Reason for exclusion
1	Akar 2014	The participants were not doctor
2	Albert 2007	The intervention was CD-ROM followed by e-mail massages
3	Aly 2012	The intervention was Internet-based
4	Ambrosio 2014	The study was not computer-based
5	Ameen 2005	The study participants were nurses
6	Backstein 2004	The intervention taught psychomotor skill
7	Backstein 2005	The intervention taught psychomotor skill
8	Bayne 1997	The study participants were nurses
9	Beckley 2000	Participants were both midwives and doctors. Results of intervention cannot be separated
10	Beidas 2012	Data could not be extracted (mixed group: doctor and therapists)
11	Benharash 2012	Data could not be extracted (mixed group: doctor and undergrade students), simulator-based intervention
12	Benjamin 2008	The intervention was web based and participants were mixed group of health professionals
13	Bhatia 2015	Protocol (ongoing study)
14	Bhatti 2011	The intervention was web-based and percipients were medical students
15	Blair 2012	The intervention was Internet-based
16	Boh, 1990	The study participants were pharmacists.
17	Boncyk 2016	Data could not be extracted, mixed group of particpant
18	Box 2011	The study evaluate psychomotor skills on simulator. Participants were mixed group:doctor and medical students
19	Brandt 2006	The study participants were medical students and the outcome was a psychomotor skill
20	Bredesen 2016	The study participants were nurses
21	Broughton 2011	The study was not RCT and the participants were nurses and recreation officers
22	Brown 2014	The intervention was Mixed, the offline computer-based effect cannot be separately evaluated

23	Browne 2004	The study was not RCT
24	Buchanan 2014	The participants were not doctor occupational therapists
25	Butzlaff 2004	The intervention was web-based
26	Campos 2011	The intervention assessed psychomotor skill
27	Chan 2013	The study was not RCT and the participants were nurses
28	Chen 1998	The participants were dental auxiliaries
29	Chiu 2009	The study participants were nurses
30	Clement 2011	The study participants were Student nurses
31	Collins 2015	Data could not be extracted, mixed group of physicians: plastic or orthopedic surgeons, and allied health professionals; physiotherapists, occupational therapists
32	Cox 2011	The participants were nurses
33	Danley 2004	Data could not be extracted, mixed group, dentists and students
34	Datta 2012	The study was not RCT
35	Davis 2008	The study participants were medical students
36	de Beurs 2015	Mixed group results for doctors cannot be extracted separately
37	de Beurs 2016	Data could not be extracted, mixed group of (nurses, psychologists, psychiatrists, & physicians)
38	Devitt 1998	Data could not be extracted, mixed group of health professionals
39	Dimeff 2011	Data could not be extracted, mixed group of health professionals
40	Dols 1995	The study participants were Nurses
41	Donyai 2015	The study participants were pharmacists.
42	Downs 2006	Data could not be extracted, mixed group of health professionals
43	Dozor 2011	The intervention was Mixed, the offline computer-based effect cannot be separately evaluated
44	Drevenhorn 2015	The study participants were nurses
45	Ebadi 2015	The study participants were nurse .
46	Edrich 2014	The intervention was web-based
47	Ekama Ilesanmi 2016	The study participants were Nurses
48	El Saadawi 2010	The intervention was Internet-based
49	Elmore 2013	The study was not RCT
50	Erkonen 1994	A long term follow up of an included study (Dallasandro)
51	Erler 2012	Mixed intervention, the offline computer-based effect cannot be separately evaluated
52	Fernandez 2013	Data for doctors could not be extracted, mixed group of health professionals
53	Fisher 2014	The study participants were nurses.
54	Friedman 1999	Participants were not randomised.

55	Gandsas 2004	The two groups interventions require a local area network
56	Gasko 2012	The study participants were student registered nurse anaesthetists
57	Gauger 2010	The intervention taught psychomotor skill
58	Gelb 2001	The study participants were medical students
59	Geller 2014	The intervention was web-based
60	Giglioli 2012	The intervention taught psychomotor skill only
61	Girgis 2009	Intervention was workshop and remote digital learning by video conference (online)
62	Glick 2012	The study participants were medical students and ED personnel intervention aims to improve psychomotor skills.
63	Gold 2004	The intervention was web-based
64	Guerra-Wallace 2010	The intervention was simulator- based
65	Hadley 2010	The intervention was web-based
66	Hamilton 2001	The intervention was simulation -based
67	Handmaker 1999	Data could not be extracted, mixed group of health professionals
68	Hansen 2015	The study not RCT
69	Harrington 2002	The study participants were nurses
70	Harrington 2003	The study participants were nurses
71	Harrington 2004	The study participants were nurses
72	Harvey 2013	The intervention was aiming at improving psychomotor skills
73	Hashimoto 2013	The intervention was virtual reality-based, and the outcome was improvement in psychomotor skills
74	Hemmati 2013	The intervention was Internet-based
75	Holmboe 2004	The intervention did not contain eLearing
76	Holmes 2012	The intervention was high- fidelity simulator-based aiming at improving psychomotor skill
77	Horn 1992	Data could not be extracted, mixed group of residents, interns and medical students
78	Hsu 2014	The study participants were nurses
79	Hymowitz 2004	The intervention was web-based
80	Hymowitz 2007	The intervention was web-based
81	Jain 2010	The intervention was web-based
82	Javadi 2015	The study participants were pharmacists.
83	Karam 2015	The intervention taught psychomotor skill
84	Khouli 2009	The study based on teaching psychomotor skills and debriefing based on performance on simulator
85	Koropchak 2006	It is a protocol (phase two study, no results)
86	Krogh 2014	The study participants were medical students

87	Kulier 2009	The intervention was Internet-based
88	Kulier 2010	The intervention was Internet-based
89	Kulier 2012	The intervention was Internet-based
90	Lange 1997	The study participants were nurses
91	Latif 2009	The intervention was simulation-based and the participants were mixed group of health professionals
92	Latzke-Davis 2011	The unit of randomizations was the practice and the study participants were physicians and nurses or medical assistants
93	Law 2003	The study was not RCT
94	Lawson 1991	The study participants were pharmacists
95	Le 2010	The intervention was web-based
96	Lee 2004	quasi-experimental, crossover design
97	Lee 2007	Data could not be extracted, mix group of health professionals
98	Lee 2016	Qualitative study, not randomised trial.
99	Leopold 2005	Study was based on teaching psychomotor skills only
100	Lieberman 2002	Data could not be extracted, mixed group of residents and medical students
101	Lin 2016	The study participants were nurses.
102	Liu 2014	The study participants were nurses
103	Loewen 2003	The study participants were mixed group of health professionals and the intervention was videoconference delivery of an education programme
104	Loveday 2010	Complex intervention including virtual reality
105	Mahmud 2013	The intervention was web-based
106	Maloney 2011	The study participants were not physicians and the intervention was web-based
107	Maloney 2012	The participants were not doctor and the intervention was web-based
108	Martinez-Escobar 2011	The intervention was based on teaching psychomotor skill only
109	Maunder 2010	Data could not be extracted, mixed group of health professionals
110	McLain 2012	The participants were student registered nurse anesthetist and the study design was a rsandomized controlled crossover trial
111	Monteiro 2016	Not digital learning
112	Moran 1991	The participants were not doctor
113	Munshi 2016	The intervention taught psychomotor skill
114	Neelankavil 2012	The intervention was simulation-based
115	Nishiyama 2009	The participants were not doctor
116	Padalino 2007	The study participants were nurses
117	Patel 2012	The intervention was Internet-based
118	Patel 2016	The intervention taught psychomotor skill

119	Perkins 2010	Data could not be extracted: mixed group of health professionals
120	Peyre 2011	The intervention was based on teaching psychomotor skill only
121	Pollak 2016	The intervention was Internet-based
122	Pusic 2007	The study participants were medical students
123	Rogers 2000	The study participants were medical students
124	Roh 2013	The study participants were nurses
125	Rosen 2002	The study participants were nurses
126	Rosenberger 2009	Data could not be extracted mixed group health practitioners.<br>A study design, no results in this paper
127	Sanddal 2004	The participants were not doctor
128	Schell 2004	The study was not RCT
129	Schermer 2011	The study participants were nurses
130	Schneider 2006	The study participants were nurses
131	Schwid 2001	The intervention was simulator based
132	Scott 2000	The intervention was based on teaching psychomotor skill only
133	Sedlack 2004	The intervention was based on teaching psychomotor skill only
134	Shariff 2012	The intervention was Internet-based
135	Shephard 2009	The study was not RCT
136	Sholomskas 2006	Data could not be extracted, mixed group of health professionals
137	Singh 2014	The study participants were medical students
138	Skinner 2009	This is a descriptive non-comparative study.
139	Smeekens 2011	The study participants were nurses
140	Sohmer 2013	The intervention was based teaching psychomotor skill only
141	Sohmer 2014	The intervention was based on teaching psychomotor skill only
142	Solomon 2004	The participants were medical students
143	Sperl-Hillen 2010	The intervention was Internet-based
144	Sperl-Hillen 2013	The intervention was Internet-based
145	Stout 2012	Data could not be extracted, mixed group of health professionals
146	Towbin 2007	The intervention was simulator- based
147	Ukabiala 2015	The full text of the abstract published under Lee et al and subsequently excluded from the review.
148	Van Es 2015	The intervention was online
149	VanderWielen 2015	Psychomoter skills learning
150	Vestal 2017	Not digital learning
151	Vollmar 2010	The intervention was Internet-based
152	Wang 2011	The intervention was simulator- based
153	Weaver 2016	Data could not be extracted (mixed group: doctor and nurses)

154	Wegener 2013	The intervention was based on teaching psychomotor skill only
155	Weiner 2011	The study participants were nurses
156	Weingardt 2006	The study participants were counsellors
157	Wetmore 2016	The outcome is improved performance which is not investigated by this review as per protocol.
158	Wilfong 2011	The study participants were nurses and the intervention was based on teaching psychomotor skills on simulator
159	Woodworth 2014	The intervention was based on teaching psychomotor skills on simulator
160	Yao 2016	Data could not be extracted (mixed group: doctor and nurses),The intervention was Internet-based
161	Zhao 2010	The intervention was simulator- based
162	Zhao 2011	The intervention was simulator- based
163	Zhao 2011a	The intervention was simulator-based
164	Bharati 2012	Abstact
165	Juyal 2013	Abstact
166	Khan 2015	Abstact
167	Lichtman 2014	Abstact
168	Muhammad 2010	Abstact

References:

1. Akar Şerife Zehra, Bebiş Hatice. Evaluation of the effectiveness of testicular cancer and testicular self-examination training for patient care personnel: intervention study. Health Education Research 2014;29(6):966-76.
2. Albert D A, Gordon J, Andrews J, Severson H, Ward A, Sadowsky D. Use of electronic continuing education in dental offices for tobacco cessation. In: Nicotine & Tobacco Research. Vol. 9. Orlando, Florida, February 2007:S137
3. Aly Medhat, Willems Guy, Van Den Noortgate Wim, Elen Jan. Effect of multimedia information sequencing on educational outcome in orthodontic training. European journal of orthodontics 2012;34(4):458-65.
4. Ambrosio Art, Pfannenstiel Travis, Bach Kevin, Cornelissen Chris, Gaconnet Cory, Brigger Matthew T. Difficult airway management for novice physicians: a randomized trial comparing direct and video-assisted laryngoscopy. Otolaryngology--head and neck

surgery : official journal of American Academy of Otolaryngology-Head and Neck Surgery 2014;150(5):775-8.

5. Ameen Jamal, Coll Anne Marie, Peters Melanie. Impact of tele-advice on community nurses' knowledge of venous leg ulcer care. Journal of advanced nursing 2005;50(6):583-94.
6. Backstein David, Agnidis Zoe, Regehr Glenn, Reznick Richard. The effectiveness of video feedback in the acquisition of orthopedic technical skills. American journal of surgery 2004;187(3):427-32.
7. Backstein David, Agnidis Zoe, Sadhu Ravi, MacRae Helen. Effectiveness of repeated video feedback in the acquisition of a surgical technical skill. Canadian journal of surgery. Journal canadien de chirurgie 2005;48(3):195-200.
8. Bayne T, Bindler R. Effectiveness of medication calculation enhancement methods with nurses. Journal of nursing staff development : JNSD 1997;13(6):293-301.
9. Beckley S, Stenhouse E, Greene K. The development and evaluation of a computer-assisted teaching programme for intrapartum fetal monitoring. BJOG 2000 Sep;107(9):1138-44.
10. Beidas Rinad S, Edmunds Julie M, Marcus Steven C, Kendall Philip C. Training and consultation to promote implementation of an empirically supported treatment: A randomized trial. Psychiatric Services 2012;63(7):660-5.
11. Benharash Peyman, Frank Paul, Hoy Brenda. Development and use of a novel cardiovascular simulator: time for a paradigm shift? The American surgeon 2012;78(10):1132-6.
12. Benjamin Sara E, Tate Deborah F, Bangdiwala Shrikant I, Neelon Brian H, Ammerman Alice S, Dodds Janice M, et al. Preparing Child Care Health Consultants to address childhood overweight: a randomized controlled trial comparing web to in-person training. Maternal and child health journal 2008;12(5):662-9.
13. Bhatia RS , Ivers N, Yin CX, Myers D, Nesbitt G, Edwards J, Yared K, Wadhera R, Wu JC, Wong B Hansen M, Weinerman A,ShadowitzS Johri A, Farkouh M, Thavendiranathan P, Udell JA, Rambihar S, Chow CM, Hall J, Thorpe KE, Rakowski H, Weiner RB. Design and methods of the Echo WISELY (Will Inappropriate Scenarios for

Echocardiography LessenSignificantlY) study: An investigator-blinded randomized controlled trial of education and feedbackintervention to reduce inappropriate echocardiograms. Am Heart J 2015;170(2):202-9.

14. Bhatti I, Jones K, Richardson L, Foreman D, Lund J, Tierney G. E-learning vs lecture: which is the best approach to surgical teaching? Colorectal disease : the official journal of the Association of Coloproctology of Great Britain and Ireland 2011;13(4):459-62.
15. Blair Irvine A, Billow Molly B, Eberhage Mark G, Seeley John R, McMahon Edward, Bourgeois Michelle. Mental illness training for licensed staff in long-term care. Issues in mental health nursing 2012;33(3):181-94.
16. Boh LE, Pitterle ME, De Muth JE. Evaluation of an Integrated Audio Cassette and Computer Based Patient Simulation Learning Program. Am J Pharm Educ 1990;54(1):15-22.
17. Boncyk CS, Schroeder KM, Anderson B, Galgon RE. Two methods for teaching basic upper airway sonography. J Clin Anesth 2016;31:166-72.
18. Box D, Shah N, Mapa B, Nagel R, Way D, Dettorre R, et al. Self-guided video review of performance does not improve learning in basic simulated tasks. Surgical Endoscopy and Other Interventional Techniques 2011;25:S285.
19. Brandt Michael G, Davies Edward T. Visual-spatial ability, learning modality and surgical knot tying. Canadian journal of surgery. Journal canadien de chirurgie 2006;49(6):412-6.
20. Bredesen IM, Bjøro K,Gunningberg L,Hofoss D. Effect of e-learning program on risk assessment and pressure ulcer classification - A randomized study. Nurse Educ Today 2016;40:191-7.
21. Broughton Megan, Smith Erin R, Baker Rosemary, Angwin Anthony J, Pachana Nancy A, Copland David A, et al. Evaluation of a caregiver education program to support memory and communication in dementia: a controlled pretest-posttest study with nursing home staff. International journal of nursing studies 2011;48(11):1436-44.
22. Brown R, Butow P, Aldridge J, Uraskova I, Zoller P, Boyle F, et al. The impact of consultation skills training in shared decision making on doctor behavior, confidence and

stress and burnout: A randomized controlled trial. In PSYCHO-ONCOLOGY 2014;23:148-9.

23. Browne L, Mehra S, Rattan R, Thomas G. Comparing lecture and e-learning as pedagogies for new and experienced professionals in dentistry. British dental journal 2004;197(2):95-7.
24. Buchanan Helen, Siegfried Nandi, Jelsma Jennifer, Lombard Carl. Comparison of an interactive with a didactic educational intervention for improving the evidence-based practice knowledge of occupational therapists in the public health sector in South Africa: a randomised controlled trial. Trials 2014;15:216.
25. Butzlaff M, Vollmar H C, Floer B, Koneczny N, Isfort J, Lange S. Learning with computerized guidelines in general practice?: A randomized controlled trial. Family practice 2004;21(2):183-8.
26. Campos Javier H, Hallam Ezra A, Ueda Kenichi. Training in placement of the left-sided double-lumen tube among non-thoracic anaesthesiologists: intubation model simulator versus computer-based digital video disc, a randomised controlled trial. European journal of anaesthesiology 2011;28(3):169-74.
27. Chan Samson, Pielak Karen, McIntyre Cheryl, Deeter Brittany, Taddio Anna. Implementation of a new clinical practice guideline regarding pain management during childhood vaccine injections. Paediatrics & child health 2013;18(7):367-72.
28. Chen M S, Horrocks E N, Evans R D. Video versus lecture: effective alternatives for orthodontic auxiliary training. British journal of orthodontics 1998;25(3):191-5.
29. Chiu Su-Chen, Cheng Kuei-Yueh, Sun Tzu-Kuan, Chang Ku-Chou, Tan Teng-Yeow, Lin Tsu-Kung, et al. The effectiveness of interactive computer assisted instruction compared to videotaped instruction for teaching nurses to assess neurological function of stroke patients: a randomized controlled trial. International journal of nursing studies 2009;46(12):1548-56.
30. Clement S, Van Nieuwenhuizen A, Kassam A, Norman I, Flach C, Lazarus A, et al. Recorded versus live social contact interventions to reduce stigma: Randomised controlled trial. Psychiatrische Praxis 2011;38(S 01):S07_4_RE.

31. Collins AM, Quinlan CS, Dolan RT, O'Neill SP, Tierney P, Cronin KJ, Ridgway PF. Audiovisual preconditioning enhances the efficacy of an anatomical dissection course: A randomised study. J Plast Reconstr Aesthet Surg 2015;68(7):1010-5.

32. Cox Jill, Roche Sharon, Van Wynen Elizabeth. The effects of various instructional methods on retention of knowledge about pressure ulcers among critical care and medical-surgical nurses. Journal of continuing education in nursing 2011;42(2):71-8.

33. Danley Dale, Gansky Stuart A, Chow Denise, Gerbert Barbara. Preparing dental students to recognize and respond to domestic violence: the impact of a brief tutorial. Journal of the American Dental Association (1939) 2004;135(1):67-73.

34. Datta A, Lema P C, Bharati A, Bluvas P, Ryan J. Resident education in ultrasonography: Improving identification of abdominal anatomy and pathology after a multimedia tutorial. Annals of Emergency Medicine 2012;60(4):S80-1.

35. Davis James, Crabb Sarah, Rogers Elizabeth, Zamora Javier, Khan Khalid. Computer-based teaching is as good as face to face lecture-based teaching of evidence based medicine: a randomized controlled trial. Medical teacher 2008;30(3):302-7.

36. de Beurs Derek P, de Groot Marieke H, de Keijser Jos, Mokkenstorm Jan, van Duijn Erik, de Winter Remco F P, et al. The effect of an e-learning supported Train-the-Trainer programme on implementation of suicide guidelines in mental health care. Journal of affective disorders 2015;175:446-53.

37. de Beurs DP, de Groot MH, de Keijser J, van Duijn E, de Winter RF, Kerkhof AJ. Evaluation of benefit to patients of training mental health professionals in suicide guidelines: cluster randomised trial. Br J Psychiatry 2016;208(5):477-83.

38. Devitt P, Worthley S, Palmer E, Cehic D. Evaluation of a computer based package on electrocardiography. Australian and New Zealand journal of medicine 1998;28(4):432-5.

39. Dimeff Linda A, Woodcock Eric A, Harned Melanie S, Beadnell Blair. Can dialectical behavior therapy be learned in highly structured learning environments? Results from a randomized controlled dissemination trial. Behavior therapy 2011;42(2):263-75.

40. Dols C, Forker J, Lumm M, Huff I, Johnson M, Morrell C, et al. Enhancing nurses' reliance on patients' perceptions of pain during pain assessment: a comparison of two educational methods. Journal of Continuing Education in Nursing 1995;26(5):209-13.

41. Donyai P, Alexander AM. Training on the use of a bespoke continuing professional development framework improves the quality of CPD records. Int J Clin Pharm 2015;37(6):1250-7.

42. Downs Murna, Turner Stephen, Bryans Michelle, Wilcock Jane, Keady John, Levin Enid, et al. Effectiveness of educational interventions in improving detection and management of dementia in primary care: cluster randomised controlled study. BMJ (Clinical research ed.) 2006;332(7543):692-6.

43. Dozor A J, Stout J W, Smith K, Zhou C, Solomon C, Mangione-Smith R. Learning A distance: A cluster randomized trial of a program to improve asthma care through interactive, online spirometry training and feedback. American Journal of Respiratory and Critical Care Medicine 2011;183(1):A5473.

44. Drevenhorn E, Bengtson A, Nyberg P, Kjellgren KI. Assessment of hypertensive patients' self-care agency after counseling training of nurses. J Am Assoc Nurse Pract 2015;27(11):624-30.

45. Ebadi A, Yousefi S, Khaghanizade M, Saeid Y. Assessment Competency of Nurses in Biological Incidents. Trauma Mon 2015;20(4):e25607.

46. Edrich T, Stopfkuchen-Evans M, Song P, Szabo A, Dankl D, Scheiermann P, et al. Comparison of web-based to traditional classroom-based training of lung ultrasound for the detection of pneumothorax. Intensive Care Medicine 2014;40(1):S203

47. Ekama Ilesanmi R, Morohunfoluwa Oluwatosin O. A Quasi-experimental Study to Assess an Interactive Educational Intervention on Nurses' Knowledge of Pressure Ulcer Prevention in Nigeria. Ostomy Wound Manage 2016;62(4):30-40.

48. Elmore B, Ginter B, McGaugh J M, Dority J. Patient simulation improves anesthesiology residents' ability to manage local anesthetic systemic toxicity as measured by written and clinical assessment. In: Regional Anesthesia and Pain Medicine. Vol. 38. 2013:354. [Other: http://www.asaabstracts.com/strands/asaabstracts/abstract.

49. El Saadawi Gilan M, Azevedo Roger, Castine Melissa, Payne Velma, Medvedeva Olga, Tseytlin Eugene, et al. Factors affecting feeling-of-knowing in a medical intelligent tutoring system: the role of immediate feedback as a metacognitive scaffold. Advances in health sciences education : theory and practice 2010;15(1):9-30.

50. Erkonen W E, D'Alessandro M P, Galvin J R, Albanese M A, Michaelsen V E. Longitudinal comparison of multimedia textbook instruction with a lecture in radiology education. Academic radiology 1994;1(3):287-92.

51. Erler Antje, Beyer Martin, Petersen Juliana J, Saal Kristina, Rath Thomas, Rochon Justine, et al. How to improve drug dosing for patients with renal impairment in primary care - a cluster-randomized controlled trial. BMC family practice 2012;13:91.

52. Fernandez R, Pearce M, Grand J A, Rench T A, Jones K A, Chao G T, et al. A randomized comparison study to evaluate the effectiveness of a computerbased teamwork training intervention on medical teamwork and patient care performance. Academic Emergency Medicine 2013;20(5):S125.

53. Fisher M J, Broome M E, Friesth B M, Magee T, Frankel R M. The effectiveness of a brief intervention or emotion-focused nurse-parent communication. Patient Education and Counseling 2014;96(1):72-8.

54. Friedman C P, Elstein A S, Wolf F M, Murphy G C, Franz T M, Heckerling P S, et al. Enhancement of clinicians' diagnostic reasoning by computer-based consultation: A multisite study of 2 systems. Journal of the American Medical Association 1999;282(19):1851-6.

55. Gandsas Alex, McIntire Katherine, Montgomery Kevin, Bumgardner Cody, Rice Linda. The personal digital assistant (PDA) as a tool for telementoring endoscopic procedures. Studies in health technology and informatics 2004;98:99-103.

56. Gasko John, Johnson Arthur, Sherner John, Craig John, Gegel Brian, Burgert James, et al. Effects of using simulation versus CD-ROM in the performance of ultrasound-guided regional anesthesia. AANA journal 2012;80(4 Suppl):S56-9.

57. Gauger Paul G, Hauge Linnea S, Andreatta Pamela B, Hamstra Stanley J, Hillard Miranda L, Arble Eamonn P, et al. Laparoscopic simulation training with proficiency targets improves practice and performance of novice surgeons. American journal of surgery 2010;199(1):72-80.

58. Gelb Douglas J. Is newer necessarily better? Assessment of a computer tutorial on neuroanatomical localization. Neurology 2001;56(3):421-2.

59. Geller Berta M, Bogart Andy, Carney Patricia A, Sickles Edward A, Smith Robert, Monsees Barbara, et al. Educational interventions to improve screening mammography interpretation: a randomized controlled trial. AJR. American journal of roentgenology 2014;202(6):W586-96.

60. Giglioli S, Boet S, De Gaudio A R, Linden M, Schaeffer R, Bould M D, et al. Self-directed deliberate practice with virtual fiberoptic intubation improves initial skills for anesthesia residents. Minerva anestesiologica 2012;78(4):456-61.

61. Girgis Afaf, Cockburn Jill, Butow Phyllis, Bowman Deborah, Schofield Penelope, Stojanovski Elizabeth, et al. Improving patient emotional functioning and psychological morbidity: evaluation of a consultation skills training program for oncologists. Patient education and counseling 2009;77(3):456-62.

62. Glick J, Leibner E, Terndrup T. Improving CPR quality in novice and experienced providers through training with an internal chest compression monitoring device. Academic Emergency Medicine 2012;19:S328.

63. Gold J P, Begg W B, Fullerton D, Mathisen D, Olinger G, Orringer M, et al. Successful implementation of a novel internet hybrid surgery curriculum - The early phase outcome of thoracic surgery Prerequisite Curriculum e-learning project. Annals of Surgery 2004;240(3):499-507.

64. Guerra-Wallace M, Zuckerbraun N S, Manole M D. nullJust-in-timenull lumbar puncture simulation training for residents in the pediatric emergency department. Pediatric Emergency Care 2010;26(9):702.

65. Hadley Julie, Kulier Regina, Zamora Javier, Coppus Sjors F P J, Weinbrenner Susanne, Meyerrose Berrit, et al. Effectiveness of an e-learning course in evidence-based medicine for foundation (internship) training. Journal of the Royal Society of Medicine 2010;103(7):288-94.

66. Hamilton E C, Scott D J, Kapoor A, Nwariaku F, Bergen P C, Rege R V, et al. Improving operative performance using a laparoscopic hernia simulator. American journal of surgery 2001;182(6):725-8.

67. Handmaker N S, Hester R K, Delaney H D. Videotaped training in alcohol counseling for obstetric care practitioners: a randomized controlled trial. Obstetrics and gynecology 1999;93(2):213-8.

68. Hansen C,Worm B,DamM,Haase N,PoulsenT,BendtsenT,Børglum J.. E-learning education for ultrasound-guided peripheral blocks. A prospective study evaluating individual increment in learning curves during a one-month trial period. In: Regional Anesthesia and Pain Medicine. Vol. 40. 2015:e110.

69. Harrington Susan S, Walker Bonnie L. A comparison of computer-based and instructor-led training for long-term care staff. Journal of continuing education in nursing 2002;33(1):39-45.

70. Harrington Susan S, Walker Bonnie L. Is computer-based instruction an effective way to present fire safety training to long-term care staff? Journal for nurses in staff development : JNSD : official journal of the National Nursing Staff Development Organization 2003;19(3):147-54.

71. Harrington Susan S, Walker Bonnie L. The effects of computer-based training on immediate and residual learning of nursing facility staff. Journal of continuing education in nursing 2004;35(4):154-7.

72. Harvey L F B, King L, Hur H C. Challenges associated with a self-contained simulation curriculum using a home laparoscopic skills trainer. Journal of Minimally Invasive Gynecology 2013;20(6):S130-1.

73. Hashimoto D A, Laura B B, Sirimanna P, Ericsson K A, Williams N N, Darzi A, et al. A randomized controlled trial to assess if deliberate practice on a virtual reality simulator can achieve "expert" surgical performance. Surgical Endoscopy and Other Interventional Techniques 2013;27:S338.

74. Hemmati Nima, Omrani Soghra, Hemmati Naser. A Comparison of Internet-Based Learning and Traditional Classroom Lecture to Learn CPR for Continuing Medical Education. Turkish Online Journal of Distance Education 2013;14(1):256-65.

75. Holmboe Eric S, Hawkins Richard E, Huot Stephen J. Effects of training in direct observation of medical residents' clinical competence: a randomized trial. Annals of internal medicine 2004;140(11):874-81.

76. Holmes J, Carleton S, Hart K, Leblanc D, Lindsell C. Sim-man vs powerpoint for teaching critical care transport medicine airway management decision- Making. Air Medical Journal 2012;31(6):257.

77. Horn D L, Radhakrishnan J, Saini S, Pepper G M, Peterson S J. Evaluation of a computer program for teaching laboratory diagnosis of acid-base disorders. Computers and biomedical research, an international journal 1992;25(6):562-8.

78. Hsu Li-Ling, Huang Ya-Hsuan, Hsieh Suh-Ing. The effects of scenario-based communication training on nurses' communication competence and self-efficacy and myocardial infarction

79. Hymowitz Norman, Schwab Joseph, Haddock Christopher Keith, Burd Karen M, Pyle Sara. The Pediatric Residency Training on Tobacco Project: baseline findings from the resident tobacco survey and observed structured clinical examinations. Preventive medicine 2004;39(3):507-16.

80. Hymowitz Norman, Schwab Joseph V, Haddock Christopher Keith, Pyle Sara A, Schwab Lisa M. The pediatric residency training on tobacco project: four-year resident outcome findings. Preventive medicine 2007;45(6):481-90.

81. Jain A, Agarwal R, Chawla D, Paul V, Deorari A. Tele-education vs classroom training of neonatal resuscitation: a randomized trial. Journal of perinatology : official journal of the California Perinatal Association 2010;30(12):773-9.

82. Javadi M, Kargar A, Gholami K, Hadjibabaie M, Rashidian A, Torkamandi H, Sarayani A. Didactic Lecture Versus Interactive Workshop for Continuing Pharmacy Education on ReproductiveHealth: A Randomized Controlled Trial. Eval Health Prof 2015;38(3):404-18.

83. Karam MD, Thomas GW, Koehler DM, Westerlind BO Lafferty PM, Ohrt GT, Marsh JL, Van Heest AE, Anderson DD. Surgical Coaching from Head-Mounted Video in the Training of Fluoroscopically Guided ArticularFracture Surgery. J Bone Joint Surg Am 2015;17;97(12):1031-9.

84. Khouli H I, Jahnes K, Mathew J, Gohil A, Shapiro J, Rose K, et al. Medical residents performance in maximum barrier precautions during central venous catheter placement:

effect of simulation-based training [Abstract]. CHEST Journal 2009;136(4_MeetingAbstracts):12s.

85. Koropchak Celine M, Pollak Kathryn I, Arnold Robert M, Alexander Stewart C, Skinner Celette Sugg, Olsen Maren K, et al. Studying communication in oncologist-patient encounters: the SCOPE Trial. Palliative medicine 2006;20(8):813-9.

86. Krogh Charlotte Loumann, Ringsted Charlotte, Kromann Charles B, Rasmussen Maria Birkvad, Todsen Tobias, Jorgensen Rasmus Lundhus, et al. Effect of engaging trainees by assessing peer performance: a randomised controlled trial using simulated patient scenarios. BioMed research international 2014;2014:610591.

87. Kulier Regina, Coppus Sjors F P J, Zamora Javier, Hadley Julie, Malick Sadia, Das Kausik, et al. The effectiveness of a clinically integrated e-learning course in evidence-based medicine: a cluster randomised controlled trial. BMC medical education 2009;9:21.

88. Kulier Regina, Khan Khalid S, Gulmezoglu A Metin, Carroli Guillermo, Cecatti Jose G, Germar Maria J, et al. A cluster randomized controlled trial to evaluate the effectiveness of the clinically integrated RHL evidence -based medicine course. Reproductive health 2010;7:8.

89. Kulier Regina, Gulmezoglu Ahmet Metin, Zamora Javier, Plana M Nieves, Carroli Guillermo, Cecatti Jose G, et al. Effectiveness of a clinically integrated e-learning course in evidence-based medicine for reproductive health training: a randomized trial. JAMA 2012;308(21):2218-25.

90. Lange L L, Haak S W, Lincoln M J, Thompson C B, Turner C W, Weir C, et al. Use of Iliad to improve diagnostic performance of nurse practitioner students. The Journal of nursing education 1997;36(1):36-45.

91. Latif R, Memon S, Bautista A, Smith E, Ziegler C, Wadhwa A. A randomized, blinded study to assess the effectiveness of simulation-based training for U/S-guided central venous access placement using aseptic technique. Critical Care Medicine 2009;37(12):A1.

92. Latzke-Davis B, Stout J, Smith K, Solomon C, Garrison M, Mangione-Smith R. Effectiveness of spirometry fundamentals(trademark) for increasing the proper use of

spirometry in patients with asthma and COPD. Primary Care Respiratory Journal 2011;20(4):441-7.

93. Law M, Hurley P, Russell D, King G, Hanna S. Selecting outcomes measures in children's rehabilitation: a comparison of methods [corrected] [published erratum appears in ARCH PHYS MED REHABIL 2004 Feb;85(2):349]. Archives of Physical Medicine & Rehabilitation 2003;84(4):496-9.

94. Lawson K A, Shepherd M D, Gardner V R. USING A COMPUTER-SIMULATION PROGRAM AND A TRADITIONAL APPROACH TO TEACH PHARMACY FINANCIAL MANAGEMENT. American Journal of Pharmaceutical Education 1991;55(3):226-35.

95. Le TT, Rait MA, Jarlsberg LG, Eid NS, Cabana MD. A randomized controlled trial to evaluate the effectiveness of a distance asthma learning program for pediatricians. The Journal of asthma : official journal of the Association for the Care of Asthma 2010;47(3):245-50.

96. Lee M T, Hishinuma E S, Derauf C, Guerrero A P S, Iwaishi L K, Kasuya R T. Smoking cessation counseling training for pediatric residents in the continuity clinic setting. Ambulatory Pediatrics 2004;4(4):289-94.

97. Lee JC, Boyd R, Stuart P. Randomized controlled trial of an instructional DVD for clinical skills teaching. Emergency medicine Australasia : EMA 2007;19(3):241-5.

98. Lee WW, Alkureishi MA, Ukabiala O, Venable LR, Ngooi SS, Staisiunas DD, Wroblewski KE, Arora VM. Patient Perceptions of Electronic Medical Record Use by Faculty and Resident Physicians: A Mixed Methods Study. J Gen Intern Med November 2016;31(11):1315-1322.

99. Leopold S S, Morgan H D, Kadel N J, Gardner G C, Schaad D C, Wolf F M. Impact of educational intervention on confidence and competence in the performance of a simple surgical task. Journal of Bone and Joint Surgery - Series A 2005;87(5):1031-7.

100. Lieberman Gillian, Abramson Richard, Volkan Kevin, McArdle Patricia J. Tutor versus computer: a prospective comparison of interactive tutorial and computer-assisted instruction in radiology education. Academic radiology 2002;9(1):40-9.

101. Lin PY, Chua WL, Mok WQ, Liaw SY. Using a case-based animation on physiological compensatory mechanism to remediate nurses'misconceptions about changes in patient vital signs. J Clin Nurs 2016;25(3-4):566-8.

102. Liu Wen- I, Rong Jiin-Ru, Liu Chieh-Yu. Using evidence-integrated e-learning to enhance case management continuing education for psychiatric nurses: a randomised controlled trial with follow-up. Nurse education today 2014;34(11):1361-7.

103. Loewen L, Seshia M M K, Fraser Askin D, Cronin C, Roberts S. Effective delivery of neonatal stabilization education using videoconferencing in Manitoba. Journal of telemedicine and telecare 2003;9(6):334-8.

104. Loveday Benjamin P T, Oosthuizen George V, Diener B Scott, Windsor John A. A randomized trial evaluating a cognitive simulator for laparoscopic appendectomy. ANZ journal of surgery 2010;80(9):588-94.

105. Mahmud Ayesha, Kettle Christine, Bick Debra, Rowley Claire, Rathod Trishna, Belcher John, et al. The development and validation of an internet-based training package for the management of perineal trauma following childbirth: MaternityPEARLS. Postgraduate medical journal 2013;89(1053):382-9.

106. Maloney Stephen, Haas Romi, Keating Jennifer L, Molloy Elizabeth, Jolly Brian, Sims Jane, et al. Effectiveness of Web-based versus face-to-face delivery of education in prescription of falls-prevention exercise to health professionals: randomized trial. Journal of medical Internet research 2011;13(4):e116.

107. Maloney Stephen, Haas Romi, Keating Jenny L, Molloy Elizabeth, Jolly Brian, Sims Jane, et al. Breakeven, cost benefit, cost effectiveness, and willingness to pay for web-based versus face-to-face education delivery for health professionals. Journal of medical Internet research 2012;14(2):e47.

108. Martinez-Escobar M, Juhnke B, Foo E, Hisley K, Lobe T, Winer E. A study evaluating the effectiveness of two-dimensional and three-dimensional medical visualization in anatomy education. Journal of Laparoendoscopic and Advanced Surgical Techniques 2011;21(4):A59.

109. Maunder Robert G, Lancee William J, Mae Reet, Vincent Leslie, Peladeau Nathalie, Beduz Mary Agnes, et al. Computer-assisted resilience training to prepare

healthcare workers for pandemic influenza: a randomized trial of the optimal dose of training. BMC health services research 2010;10:72.

110. McLain N E, Biddle C, James Cotter J. Anesthesia clinical performance outcomes: Does teaching method make a difference? AANA Journal 2012;80(SUPPL.4):S11-6.

111. RS Monteiro, PM Letchworth, JG Evans, MJ Anderson, SP Duff. Obstetric emergency training in a rural South African DGH –simple measures improve knowledge. In: International Journal of Obstetric Anesthesia. Vol. 25. May 2016:S35. [DOI: 10.1016/j.ijoa.2016.02.005; anaes.ac.uk/assets/_managed/cms/files/Meeting%20Docs/abstracts/2016/2016_IJOA_

112. Moran M L. Hypertext computer-assisted instruction for geriatric physical therapists. Physical and Occupational Therapy in Geriatrics 1991;10(2):31-53.

113. MunshiA. G,WatanabeY,McKendy K,ItoY.M ,Fried G. M,Vassiliou M. C,Feldman L. S. Use of a formative feedback tool in place of an expert coach in laparoscopic suturing training: A randomised non-inferiority trial. Surgical Endoscopy and Other Interventional Technique 2016;30:S359.

114. Neelankavil Jacques, Howard-Quijano Kimberly, Hsieh Tyken C, Ramsingh Davinder, Scovotti Jennifer C, Chua Jason H, et al. Transthoracic echocardiography simulation is an efficient method to train anesthesiologists in basic transthoracic echocardiography skills. Anesthesia and analgesia 2012;115(5):1042-51.

115. Nishiyama C, Iwami T, Kawamura T, Ando M, Kajino K, Yonemoto N, et al. Effectiveness of simplified chest compression-only CPR training program with or without preparatory self-learning video: A randomized controlled trial. Resuscitation 2009;80(10):1164-8.

116. Padalino Yara, Peres Heloisa Helena Ciqueto. E-learning: a comparative study for knowledge apprehension among nurses. Revista latino-americana de enfermagem 2007;15(3):397-403.

117. Patel Vishal, Aggarwal Rajesh, Osinibi Elizabeth, Taylor Dave, Arora Sonal, Darzi Ara. Operating room introduction for the novice. American journal of surgery 2012;203(2):266-75.

118. Patel N. R,Makai G. E,Sloan N. L,Della Badia C. R. Traditional Versus Simulation Resident Surgical Laparoscopic Salpingectomy Training: A Randomized Controlled Trial. Journal of minimally invasive gynecology 2016;23(3):372-377.

119. Perkins Gavin D, Fullerton James N, Davis-Gomez Nicole, Davies Robin P, Baldock Catherine, Stevens Harry, et al. The effect of pre-course e-learning prior to advanced life support training: a randomised controlled trial. Resuscitation 2010;81(7):877-81.

120. Peyre S E, West A, Pernar L, Hicks G, Smink D. A randomized control study of the effectiveness of video instruction for the acquisition of basic laparoscopic surgical skill. Journal of the American College of Surgeons 2011;213(3):S128.

121. Pollak KI, Tulsky JA, Bravender T, Østbye T, Lyna P, Dolor RJ, Coffman CJ, Bilheimer A, Lin PH, Farrell D, Bodner ME, Alexander SC. Teaching primary care physicians the 5 A's for discussing weight with overweight and obeseadolescents.. Patient Educ Couns 2016;99(10):1620-5.

122. Pusic Martin V, Pachev George S, MacDonald Wendy A. Embedding medical student computer tutorials into a busy emergency department. Academic emergency medicine : official journal of the Society for Academic Emergency Medicine 2007;14(2):138-48.

123. Rogers D A, Regehr G, Gelula M, Yeh K A, Howdieshell T R, Webb W. Peer teaching and computer-assisted learning: An effective combination for surgical skill training? The Journal of surgical research 2000;92(1):53-5.

124. Roh Young Sook, Lee Woo Sook, Chung Hyun Soo, Park Young Mi. The effects of simulation-based resuscitation training on nurses' self-efficacy and satisfaction. Nurse education today 2013;33(2):123-8.

125. Rosen Jules, Mulsant Benoit H, Kollar Marcia, Kastango Kari B, Mazumdar Sati, Fox Debra. Mental health training for nursing home staff using computer-based interactive video: a 6-month randomized trial. Journal of the American Medical Directors Association 2002;3(5):291-6.

126. Rosenberger Erica L, Goff David C Jr, Blackwell Caroline S, Williams Dustin T, Crago O Lenore, Ellis Shellie D, et al. Implementing a palm pilot intervention for primary care providers: lessons learned. Contemporary clinical trials 2009;30(4):321-5.

127. Sanddal Nels D, Sanddal Teri L, Pullum Jeri D, Altenhofen Katrina B, Werner Susan M, Mayberry James, et al. A randomized, prospective, multisite comparison of pediatric prehospital training methods. Pediatric emergency care 2004;20(2):94-100.

128. Schell Scott R, Flynn Timothy C. Web-based minimally invasive surgery training: competency assessment in PGY 1-2 surgical residents. Current surgery 2004;61(1):120-4.

129. R, Akkermans Reinier P, Crockett Alan J, van Montfort Marian, Grootens-Stekelenburg Joke, Stout Jim W, et al. Effect of e-learning and repeated performance feedback on spirometry test quality in family practice: a cluster trial. Annals of family medicine 2011;9(4):330-6.

130. Schneider Philip J, Pedersen Craig A, Montanya Kathryn R, Curran Christine R, Harpe Spencer E, Bohenek Wayne, et al. Improving the safety of medication administration using an interactive CD-ROM program. American journal of health-system pharmacy : AJHP : official journal of the American Society of Health-System Pharmacists 2006;63(1):59-64.

131. Schwid H A, Rooke G A, Michalowski P, Ross B K. Screen-based anesthesia simulation with debriefing improves performance in a mannequin-based anesthesia simulator. Teaching and learning in medicine 2001;13(2):92-6.

132. Scott D J, Rege R V, Bergen P C, Guo W D A, Laycock R, Tesfay S T, et al. Measuring operative performance after laparoscopic skills training: Edited videotape versus direct observation. Journal of Laparoendoscopic & Advanced Surgical Techniques-Part A 2000;10(4):183-90.

133. Sedlack Robert E, Kolars Joseph C. Computer simulator training enhances the competency of gastroenterology fellows at colonoscopy: results of a pilot study. The American journal of gastroenterology 2004;99(1):33-7.

134. Shariff U, Kullar N, Haray P N, Dorudi S, Balasubramanian S. Multimedia educational tool for cognitive surgical skill acquisition in open and laparoscopic colorectal surgery. Colorectal Disease 2012;14:18.

135. Shephard Mark D, Mazzachi Beryl C, Watkinson Les, Shephard Anne K, Laurence Caroline, Gialamas Angela, et al. Evaluation of a training program for device operators in the Australian Government's Point of Care Testing in General Practice Trial: issues and implications for rural and remote practices. Rural and remote health 2009;9(3):1189.

136. Sholomskas Diane E, Carroll Kathleen M. One small step for manuals: Computer-assisted training in twelve-step facilitation. Journal of studies on alcohol 2006;67(6):939-45.

137. Singh P, Aggarwal R, Tahir M, Pucher P H, Darzi A. A randomized controlled study to evaluate the role of video-based coaching in training laparoscopic skills. Annals of Surgery 2014;261(5):862-869.

138. Skinner Celette Sugg, Pollak Kathryn I, Farrell David, Olsen Maren K, Jeffreys Amy S, Tulsky James A. Use of and reactions to a tailored cd-rom designed to enhance oncologist-patient communication: The scope trial intervention. Patient Education and Counseling 2009;77(1):90-6.

139. Smeekens A E F N, Broekhuijsen-van Henten D M, Sittig J S, Russel I M B, ten Cate O Th J, Turner N M, et al. Successful e-learning programme on the detection of child abuse in emergency departments: a randomised controlled trial. Archives of disease in childhood 2011;96(4):330-4.

140. Sohmer B, Hudson C, Hudson J, Posner G, Naik V. Psychomotor readiness of beginner echocardiographers nullprobenull. Canadian Journal of Anesthesia 2013;60(1):S42.

141. Sohmer B, Hudson C, Hudson J, Posner G D, Naik V. Transesophageal echocardiography simulation is an effective tool in teaching psychomotor skills to novice echocardiographers. Canadian Journal of Anesthesia 2014;61(3):235-41.

142. Solomon David J, Ferenchick Gary S, Laird-Fick Heather S, Kavanaugh Kevin. A randomized trial comparing digital and live lecture formats [ISRCTN40455708. BMC medical education 2004;4:27.

143. Sperl-Hillen J M, O'Connor P J, Rush W A, Johnson P E, Gilmer T, Biltz G, et al. Simulated physician learning program improves glucose control in adults with diabetes. Diabetes care 2010;33(8):1727-1733.

144. Sperl-Hillen J A, O'Connor P, Rush W, Ekstrom H, Appana D, Fernandes O, et al. Simulated provider training improves diabetes management. Journal of Diabetes Science and Technology 2013;7(1):A130.

145. Stout James W, Smith Karen, Zhou Chuan, Solomon Cam, Dozor Allen J, Garrison Michelle M, et al. Learning from a distance: effectiveness of online spirometry training in improving asthma care. Academic pediatrics 2012;12(2):88-95.

146. Towbin Alexander J, Paterson Brian, Chang Paul J. A computer-based radiology simulator as a learning tool to help prepare first-year residents for being on call. Academic radiology 2007;14(10):1271-83.

147. Obiama Ukabiala, Wei Wei Lee, Lollita Alkureishi, Laura Ruth Venable, Samantha Ngooi,Jeanne M.Farnan Vineet M. Arora. I JUST WANT MY DOCTOR'S UNDIVIDED ATTENTION": PATIENT PERCEPTIONS OF THE IMPACT OF EMR USE ON COMMUNICATION. Journal of General Internal Medicine 2015;30(Suppl 2):45.

148. VanderWielen BA, Harris R, Galgon RE, VanderWielen LM, Schroeder KM. Teaching sonoanatomy to anesthesia faculty and residents: utility of hands-on gel phantom and instructional video training models. J Clin Anesth 2015;27(3):188-94.

149. Van Es SL, Pryor WM, Belinson Z, Salisbury EL, Velan GM. Cytopathology whole slide images and virtual microscopy adaptive tutorials: A software pilot.. J Pathol Inform. 2015 28;6:54;6:54.

150. Vestal HS, Sowden G, Nejad S, Stoklosa J, Valcourt SC, Keary C, Caminis A, Huffman J. Simulation-Based Training for Residents in the Management of Acute Agitation: A Cluster Randomized Controlled Trial. Acad Psychiatry 2017;41(1):62-67.

151. Vollmar H C, Mayer H, Ostermann T, Butzlaff M E, Sandars J E, Wilm S, et al. Knowledge transfer for the management of dementia: a cluster-randomised trial of blended learning in general practice. Implementation Science 2010;5:10.

152. Wang Carolyn L, Schopp Jennifer G, Petscavage Jonelle M, Paladin Angelisa M, Richardson Michael L, Bush William H. Prospective randomized comparison of standard didactic lecture versus high-fidelity simulation for radiology resident contrast reaction management training. AJR. American journal of roentgenology 2011;196(6):1288-95.

153. Weaver MR, Pillay E, Jed SL, de Kadt J, Galagan S, Gilvydis J, Marumo E, Mawandia S, Naidoo E, Owens T, Prongay V, O'Malley G. Three methods of delivering clinic-based training on syndromic management of sexually transmitteddiseases in South Africa: a pilot study. Sex Transm Infect 2016;92(2):135-41.

154. Wegener Jessica T, van Doorn C Thea, Eshuis Jan H, Hollmann Markus W, Preckel Benedikt, Stevens Markus F. Value of an electronic tutorial for image interpretation in ultrasound-guided regional anesthesia. Regional anesthesia and pain medicine 2013;38(1):44-9.

155. Weiner Gary M, Menghini Karin, Zaichkin Jeanette, Caid Ann E, Jacoby Carrie J, Simon Wendy M. Self-directed versus traditional classroom training for neonatal resuscitation. Pediatrics 2011;127(4):713-9.

156. Weingardt Kenneth R, Villafranca Steven W, Levin Cindy. Technology-based training in cognitive behavioral therapy for substance abuse counselors. Substance abuse : official publication of the Association for Medical Education and Research in Substance Abuse 2006;27(3):19-25.

157. Wetmore D, Goldberg A, Gandhi N, Spivack J, McCormick P, DeMaria S Jr. An embedded checklist in the Anesthesia Information Management System improves pre-anaesthetic induction setup: a randomised controlled trial in a simulation setting. BMJ Qual Saf 2016;25(10):739-46.

158. Wilfong D N, Falsetti D J, McKinnon J L, Daniel L H, Wan Q. The effects of virtual intravenous and patient simulator training compared to the traditional approach of teaching nurses. Journal of Infusion Nursing 2011;34(1):55-62.

159. Woodworth, G. E., Chen, E. M., Horn, J. L. E., & Aziz, M. F.. Efficacy of computer-based video and simulation in ultrasound-guided regional anesthesia training.. Journal of clinical anesthesia 2014;26(3):212-221.

160. Yao K1, Uedo et alDevelopment of an E-learning System for the Endoscopic Diagnosis of Early Gastric Cancer: AnInternational Multicenter Randomized Controlled Trial. EBio Medicine 2016;9:140-7.

161. Zhao Y C, Hall R, Kennedy G, Leary S J O'. Does virtual reality simulation training improve real life performances? Results from a randomised control trial [Abstract]. Anz Journal of Surgery. 2010;80(s1):A80.

162. Zhao Yi C, Kennedy Gregor, Yukawa Kumiko, Pyman Brian, O'Leary Stephen. Can virtual reality simulator be used as a training aid to improve cadaver temporal bone dissection? Results of a randomized blinded control trial. The Laryngoscope 2011;121(4):831-7.

163. Zhao Yi Chen, Kennedy Gregor, Yukawa Kumiko, Pyman Brian, O'Leary Stephen. Improving temporal bone dissection using self-directed virtual reality simulation: results of a randomized blinded control trial. Otolaryngology--head and neck surgery : official journal of American Academy of Otolaryngology-Head and Neck Surgery 2011;144(3):357-64.

164. Bharati A, Datta A, Gupta S, Chun-Lema P, Ryan J. Resident education in ultrasonography: Assessment of identification of basic cardiac anatomy and pathology after a multimedia tutorial. Annals of Emergency Medicine 2012;60(4):S41.

165. Juyal M, Ware A, Houk J, Mina R. Electronic learning module enhances rheumatology education. Arthritis and Rheumatism 2013;65:S411.

166. KhanT,Cinnor B, GuptaN,Hosford L,Bansal A,Olyaee M, Wani S,Rastogi A. Comparison of classroom didactic teaching (DT) and computer based self-learning (Sl) in recognizing narrow band imaging (NBI) patterns for diminutive polyp (DP) histology characterization. Gastrointestinal Endoscopy 2015;81(5):AB325.

167. Lichtman A S, Goff B, Schreuder H W R, Lenihan Jr J, Lefebvre G, Parker W, et al. A validation study: Does use of an interactive computer based laparoscopic hysterectomy trainer expand cognitive surgical knowledge of ob/gyn trainees beyond that of traditional educational tools? Journal of Minimally Invasive Gynecology 2014;21(6):S87.

168. Muhammad A, Nutland S, Unsworth J. Electronic vs. standard learning in hospital trust induction for junior doctors - a randomized trial. British Journal of Surgery 2010;97:49-50.
