# Supplementary material for: Medical Doctors’ Offline Computer-Assisted Digital Education: Systematic Review by the Digital Health Education Collaboration
Source: J Med Internet Res. 2019 Mar 1;21(3):e12998. doi: 10.2196/12998 (PMC6418481; doi:10.2196/12998)
Supplement: Multimedia Appendix 3 [file jmir_v21i2e12998_app3.pdf]

**Author (year) Country**	**Setting/ Medical discipline/ Participants**	**Trial Characteristics**	**Risk of Bias**	**Outcome assessed**	**Results**
**Offline computer-based digital learning *vs* Face-to-face learning**					
Boet 2010 [48] Canada	Setting: University simulation center<br>Medical discipline: Anesthesia<br>Participants: 50 Postgraduate residents	Study design: Parallel RCT<br>IG: Watched offline video for self-debriefing following management of simulation crisis.<br>CG: Received instructor-debriefing following management of simulation crisis.<br>Technology type/Duration: Video recording/ 30-min pre- intervention, 20 min debriefing, 5 min duration of the scenarios<br>Instruments: Valid and reliable tool; Anesthetists' Non-Technical Skills (ANTS), Cronbach α (0.79 to 0.86)<br>Source of funding: The Network of Excellence in Simulation for Clinical Teaching & Learning, Toronto, Canada.<br>Conflict of interest: Not reported	High risk	Cognitive skill gain	Pre-intervention Mean ± SD: IG:11.36±2.18, CG:11.80±2.19<br>Post-intervention Mean ± SD: IG:12.44±1.93, CG:13.27±1.33<br>Absolute change from the baseline: IG:1.08, CG:1.47<br>Post-intervention Ratio of the mean (IG/CG): 0.94<br>P value: 0.58<br><br>Conclusion: No difference in the outcome between the two groups
D'Allessandro 1993 [43] USA	Setting: University Hospital	Study design: Parallel RCT<br>IG: Received a multimedia textbook	High risk	Cognitive skill gain	Pre-intervention Mean ± SD: IG: 58.8, CG: 53.3

	Medical discipline: Radiology<br>Participants: 49 Residents and faculty	imaging of diffuse lung disease (DLD).<br>CG: Received 50-minute lecture on the same material (DLD) as in multimedia textbook.<br>Technology type/Duration: Multimedia/ Not reported<br>Instruments: low internal validity questionnaire to assess knowledge and cognitive skills together (10 questions; 3 of the 10 questions were on image pattern recognition (cognitive skills), and 7 questions on knowledge)<br>Source of funding: Not reported<br>Conflict of interest: Not reported		Knowledge gain	Post-intervention Mean ± SD: IG:83.2±29.56, CG:82.9±35.05<br>Absolute change from the baseline: IG:24.4, CG:29.6<br>Post-intervention Ratio of the mean (IG/CG): 1<br>P value: 0.28<br><br>Conclusion: No difference in the outcome between the two groups
Davis 2007 [55] UK	Setting: Postgraduate education and training center<br>Medical discipline: Evidence-based medicine<br>Participants: 55 Medical interns	Study design: Parallel RCT<br>IG: Received computer-based (CD-ROM) sessions on evidence-based medicine (EBM) and systematic reviews.<br>CG: Received face-to-face lecture-based sessions on EBM and systematic reviews with same content.<br>Technology type/Duration: Software or computer-based programs/ 40	Low risk	Knowledge gain	Pre-intervention Mean ± SD: IG: 11.1±2.2, CG: 10.0±3.0<br>Post-intervention Mean ± SD: IG:13.2±2.54, CG: 11.9±2.54<br>Absolute change from the baseline: IG:2.1, CG:1.9<br>Post-intervention Ratio of the mean (IG/CG): 1.11<br>P value: 0.06

		min and 10 min for each questionnaire<br>Instruments: Validated assessment tools, included 5 questions on knowledge. (Two structured questions and three MCQs).<br>Source of funding: Health Education England (West Midlands Deanery<br>Conflict of interest: No conflict of interest			Conclusion: No difference in the outcome between the two groups
Esfahani 2014 [61]<br>Iran	Setting: University Hospital<br>Medical discipline: Psychiatry<br>Participants: 14 Postgraduate Residents	Study design: Parallel RCT<br>IG: Residents were given one day workshop and videotape in communication skills.<br>CG: Residents were given two days workshops in communication skills.<br>Technology type/Duration: Video recording /2-day workshop<br>Instruments: Validated and reliable tool; Jefferson's empathy scale, (measure the perception of physician empathy) AND Invalidated tool; the objective assessment of empathy rated by two certified psychiatrists independently.<br>Source of funding: Iranian Mental Health Research Network.<br>Conflict of interest: Not reported	High risk	Cognitive skill gain	Pre-intervention Mean ± SD: IG: 116.3 ±11.2, CG: 122.7±4.8<br>Post-intervention Mean ± SD: IG:114.3±14.1, CG:125±7.9<br>Absolute change from the baseline: IG: -2, CG:2.3<br>Post-intervention Ratio of the mean (IG/CG): 0.91<br>P value: <0.001<br><br>Conclusion: Face-to-face (control) group had better outcome
Farahmand 2016 [62]<br>Iran	Setting: University Hospital	Study design: Parallel RCT<br>IG: Interns received DVD consisted of 35	High risk	Cognitive skill gain	Pre-intervention Mean ± SD: NR<br>Post-intervention Mean ± SD: IG:

	Medical discipline: ALS course (trauma life support)<br>Participants: 120 Medical interns	minutes lecture, 15 minutes simulated scenario on initial management of trauma patients then hands on session on mannequin for 50 minutes.<br>CG: Interns received trauma workshop composed of 35 minutes lecture, 15 minutes case simulation by the same contents and instructor followed also by hands on session on mannequin for 50 minutes.<br>Technology type/Duration: Software or computer-based programs/ 100 min<br>Instruments: Validated tool: OSCE<br>Source of funding: Educational Development Center of Tehran University of Medical Sciences (TUMS)<br>Conflict of interest: Not reported			16.5 ± 1.96, CG: 12.3 ± 2.22<br>Absolute change from the baseline: NR<br>Post-intervention Ratio of the mean (IG/CG): 1.34<br>P value: 0.001<br><br>Conclusion: OCDE group had better outcome
Farokhi 2016 [63] Iran	Setting: Conducted in Fars Province, Iran by psychiatrists who participated in the addiction detoxification continuous training program<br>Medical discipline: Psychiatry	Study design: Parallel RCT<br>IG: electronic learning method on addiction detoxification designed in the interactive multimedia form (a professor's voice, related images and animations, clinical specimens) with self-assessment training at the end of each segment. the content was made available on compact disc to the research subjects. The	High risk	Knowledge gain	Pre-intervention Mean ± SD: IG: 13.15 ±1.62, CG: 13.25±1.87<br>Post-intervention Mean ± SD: IG: 18.84 ±1.61, CG:17.06 ±1.83<br>Absolute change from the baseline: IG:5.69, CG:3.81<br>Post-intervention Ratio of the mean (IG/CG): 1.1<br>P value: <0.01

	Participants: 118 Medical interns	subjects after studying the interactive content and passing all sections of the course proceeded to the posttest section of the compact disc.<br>CG: A professor taught lessons using lectures and power point presentations along with questions and answers.<br>Technology type/Duration: Software or computer based programs/ IG: not specified, CG: 12 hours<br>Instruments: Validated and reliable 24 MCQs (closed and regular questions)<br>Source of funding: None<br>Conflict of interest: No conflict of interest			Conclusion: OCDE group had better outcome
Isaranuw-atchai 2016 [51] Canada	Setting: University simulation center<br>Medical discipline: Anesthesia<br>Participants: 50 Postgraduate Residents (the same sample included in the Boet 2010 [48]	Study design: Parallel RCT/Cost effectiveness analysis<br>IG: Watched offline video for self-debriefing following management of simulation crisis.<br>CG: Received instructor-debriefing following management of simulation crisis.<br>Technology type/Duration: Video recording / 30-min before intervention (pre). 20 min debriefing .5 min duration of the scenarios (post)<br>Source of funding: Ottawa Hospital	High risk	Cost-effectiveness	Pre-intervention Mean ± SD: NA<br>Post-intervention Mean ± SD: NA<br>Absolute change from the baseline: NA<br>Post-intervention Ratio of the mean (IG/CG): NA<br>P value: NA<br><br>Conclusion: OCDE was cost-effective when compared to face-to-face learning if the cost of the intervention was ≤ Can$ 200

		Anesthesia Alternate Funds Association<br>Conflict of interest: No conflict of interest			
Farrar 2008 [41] Australia	Setting: University Hospital<br>Medical discipline: Pediatrics<br>Participants: 66 Practicing doctors	Study design: Quasi-RCT<br>IG: Participant worked individually on interactive teaching module presented on CD-ROM (Computerized tutorial) to learn about childhood epilepsy (i.e. diagnostic approach to a paroxysmal event).<br>CG: Facilitated case discussion tutorial with same content.<br>Technology type/Duration: Software or computer-based programs/ IG:20-30 min, CG:45 min<br>Instruments: Invalidated anonymous scoring by the authors compared with a gold standard prepared by 3 neurologists<br>Source of funding: Sydney Children's Hospital Foundation.<br>Conflict of interest: Not reported	High risk	Knowledge gain	Pre-intervention Mean ± SD: IG: 49.39±NR, CG: 50.30±NR<br>Post-intervention Mean ± SD: IG:63.91± NR, CG:54.82± NR<br>Absolute change from the baseline: IG:14.52, CG:4.52<br>Post-intervention Ratio of the mean (IG/CG): 1.17<br>P value: <0.01<br><br>Conclusion: OCDE group had better outcome
Garcia-Rodriguez 2016 [49] Canada	Setting: University Hospital<br>Medical discipline: General Practice/Family Medicine<br>Participants: 39 Postgraduate Residents	Study design: Parallel RCT<br>IG: 10 min video-module to teach the IUD insertion procedure.<br>CG: Face-to-Face traditional approach to IUD instruction with same content and instructions.	Unclear risk	Knowledge gain	Pre-intervention Mean ± SD: IG: 9.0±2.33, CG: 8.0±2.52<br>Post-intervention Mean ± SD: IG:12.3±1.87, CG:11.3 ± 1.70<br>Absolute change from the baseline: IG: 3.3, CG:3.3

		Technology type/Duration: Video recording/ 10 min<br>Instruments: Invalidated seven questions with one to three items each that required to provide short written responses. The answers for these questions were scored on a scale from 0 to 2 (0=incomplete answer, 1=partial answer, 2=complete answer. Maximum score: 14).<br>Source of funding: None<br>Conflict of interest: Not reported			Post-intervention Ratio of the mean (IG/CG): 1.0<br>P value: > 0.05<br><br>Conclusion: No difference in the outcome between the two groups
Pelayo 2000 [60] Spain	Setting: Family and community medicine teaching unit<br>Medical discipline: General Practice /Family Medicine<br>Participants: 40 Postgraduate Residents	Study design: Parallel RCT<br>IG: Computer interactive program on clinical research methodology and hand out material<br>CG: Face-to-Face on clinical research methodology and written material with same content and distribution as IG.<br>Technology type/Duration: Software or computer based programs / 2-3 hours<br>Instruments: Invalidated tests contained true/false responses, with some simple exercises.<br>Source of funding: Fondo- de Invstigacion Sanitaria [57] of Spain`s Ministry of Health and Consumer Affairs	High risk	Knowledge gain	Pre-intervention Mean ± SD: NR<br>Post-intervention Mean ± SD: IG:99.60±9.67, CG:88.17±8.45<br>Absolute change from the baseline: NR<br>Post-intervention Ratio of the mean (IG/CG): 1.13<br>P value: 0.0003<br><br>Conclusion: OCDE group had better outcome

		Conflict of interest: Not reported			
Welke 2009 [54] Canada	Setting: University Hospital Medical discipline: Anesthesia Participants: 30 Postgraduate Residents	Study design: Parallel RCT IG: Computer-based multimedia program (a standardized computer-based multimedia debriefing on crisis management of three cardiac arrest scenarios) CG: Face-to-Face (traditional personalized video-assisted oral debriefing on crisis management of three cardiac arrest scenarios). Technology type/Duration: Multimedia/ 10 min each scenario followed by debriefing Instruments: Reliable and validated tool (ANTS), Cronbach α (0.79 to 0.86) Source of funding: The Physician Services Incorporated Foundation, North York, Ontario, Canada. Conflict of interest: Not reported	Low risk	Cognitive skill gain	Pre-intervention Mean ± SD: IG:10.27±2.10, CG:9.00±2.45 Post-intervention Mean ± SD : IG:12.22±2.19, CG:11.3±2.08 Absolute change from the baseline: IG:1.95, CG:2.3 Post-intervention Ratio of the mean (IG/CG): 1.08 P value: 0.97<br><br>Conclusion: No difference in the outcome between the two groups
Platz 2011 [47] USA	Setting: Teaching hospital Medical discipline: Surgery Participants: 44 Postgraduate Residents	Study design: Parallel RCT IG: Computer based lecture on image recognition and interpretation (i.e. basic ultrasound and the extended focused assessment with sonography for trauma (EFAST)). the Computer group listened to narrated lectures on desktop computers ends with	High risk	Cognitive skill gain<br><br>Knowledge gain	Pre-intervention Mean ± SD: IG:63.2 ±, CG:58.0 Post-intervention Mean ± SD: IG:81.6 ± 20.75, CG:85.9 ± 31.38 Absolute change from the baseline: IG:18.4, CG:28.0 Post-intervention Ratio of the mean (IG/CG): 0.95 P value: <0.05

		question-and-answer session.<br>CG: Face-to-Face lectures on image recognition and interpretation with same content, and the same instructor who narrated the computer-based lectures.<br>Technology type/Duration: Software or computer based programs/ One hour<br>Instruments: Invalidated 20 items MCQs (knowledge and cognitive)<br>Source of funding: Department of Emergency Medicine, Birmingham and Women's Hospital, Boston, Massachusetts.<br>Conflict of interest: Not reported			Conclusion: Face-to-face (control) group had better outcome
Hards 2012 [50] Canada	Setting: University Hospital<br>Medical discipline: Anesthesia<br>Participants: 20 Postgraduate Residents	Study design: Parallel RCT<br>IG: The participants were pre-tested using high-fidelity simulation on management of maternal cardiac arrest, followed by computerized digital learning session, Posttest was done by simulation after one month.<br>CG: The participants were tested using high-fidelity simulation, then 30-minute narrative-based power point-based teaching session followed by discussion. Posttest was done by	High risk	Cognitive skill gain	Pre-intervention Mean ± SD: IG: 2.7±0.8, CG: 2.1±0.8<br>Post-intervention Mean ± SD: IG:3.4±0.6, CG:2.9±0.5<br>Absolute change from the baseline: IG:0.70, CG:0.80<br>Post-intervention Ratio of the mean (IG/CG): 1.17<br>P value: 0.97<br><br>Conclusion: No difference in the outcome between the two groups

		simulation after one month.<br>Technology type/Duration: Software or computer based programs / IG: own pace and convenience. CG: a 30-min, narrated Power-Point-based teaching session<br>Instruments: Reliable and validated tool (ANTS), Cronbach α (0.79 to 0.86)<br>Source of funding: Ontario Ministry of Health and Long-term Care, Ontario, Canada.<br>Conflict of interest: No conflict of interest			
Khoshbaten 2014 [64]<br>Iran	Setting: University Hospital<br>Medical discipline: ALS course (cardio-pulmonary resuscitation)<br>Participants: 84 Medical interns	Study design: Parallel RCT<br>IG: interns Received pretest, then blended learning (CD and traditional face-to-face) then a posttest. The eLearning composed of electronic software on indications, contraindications, drug dosage, and precautionary principles of 45 Cardio-pulmonary resuscitation (CPR) drugs, plus an 11-minute educational clip on ACLS including usage of drugs.<br>CG: Received a pretest, then a six-hour lecture-based educational course for the use of 45 CPR) drugs, followed by posttest.	High risk	Knowledge gain	Pre-intervention Mean ± SD: IG:7.58±2.5, CG:6.97±2.25<br>Post-intervention Mean ± SD: IG:11.87±3.66, CG:10.44±3.68<br>Absolute change from the baseline: IG:4.29, CG:3.47<br>Post-intervention Ratio of the mean (IG/CG): 1.13<br>P value: 0.49<br><br>Conclusion: No difference in the outcome between the two groups

		Technology type/Duration: Software or computer based programs/ IG: one month, CG: six-hour<br>Instruments: Validated and reliable tool of 21 MCQs<br>Source of funding: Medical Education Research Center, Tabriz University of Medical Sciences, Iran<br>Conflict of interest: No conflict of interest			
Tulsky 2011 [53] Canada	Setting: University Hospital<br>Medical discipline: Oncology<br>Participants: 48 Practicing doctors	Study design: Parallel stratified RCT<br>IG: The oncologists received a CD-ROM training program on communication skills also received complete audio recordings of each of their recorded patient visits.<br>CG: The oncologists received no training beyond the 1-hour lecture.<br>Technology type/Duration: Software or computer-based programs/ One month<br>Instruments: Reliable Tool: Empathy assessment count using NURSE (name, understand, respect, support and explore) by review audiotape recording. (Cognitive skills). Reliable tool: Patient perceptions were measured during patient interview. (Patient outcome)	High risk	Cognitive skill gain	Pre-intervention Mean ± SD: NR<br>Post-intervention Mean ± SD: IG: RR, 0.7 95% CI (0.5 to 1.0), CG: RR, 0.495% CI (0.3 to 0.5)<br>Absolute change from the baseline: NR<br>Post-intervention Ratio of the mean (IG/CG): NR<br>P value: 0.024<br><br>Conclusion: OCDE group had better outcome

		Source of funding: National Cancer Institute.<br>Conflict of interest: No conflict of interest		Patient's outcome	Pre-intervention Mean ± SD: NR<br>Post-intervention Mean ± SD: IG: 4.7 95% CI (4.6 to 4.8), CG: 4.6 95% CI (4.5 to 4.7)<br>Absolute change from the baseline: NR<br>Post-intervention Ratio of the mean (IG/CG): NR<br>P value: 0.036<br><br>Conclusion: No difference in the outcome between the two groups
Millard 2008 [40] Australia	Setting: University Hospital<br>Medical discipline: General Practice /Family Medicine<br>Participants: 14 doctors	Study design: Factorial RCT, only two arms were included in this review<br>IG: Received education (workshop) on dementia diagnoses documentation in addition to audit on their dementia risk factors recognition and diagnosis documentation during the 2 year prior to the intervention.<br>CG: Received education only.<br>Technology type/Duration: Software or computer-based programs/ 2 hours<br>Instruments: Invalidated auditing generated by computer (improvement of dementia diagnosis and screening documentation)<br>Source of funding: Not reported	High risk	Patient's outcome	Pre-intervention Mean ± SD: NR<br>Post-intervention Mean ± SD: IG:13.25 (ranked mean), CG: 6.13 (ranked mean)<br>Absolute change from the baseline: IG: 6.1 (mean difference)a, CG: 1.0 (mean difference)a<br>Post-intervention Ratio of the mean (IG/CG): NR<br>P value: 0.018<br><br>Conclusion: OCDE group had better outcome

		Conflict of interest: Not reported			
**Offline computer-based digital learning vs No intervention**					
Garrett 1990 [44] USA	Setting: University Hospital Medical discipline: Internal Medicine Participants: 57 Postgraduate Residents	Study design: Parallel RCT IG: computerized assistant instruction on AIDS Infection control. CG: Routine training on AIDS Infection control. Technology type/Duration: Software or computer-based programs/ Not reported Instruments: Invalidated 48 test of True/False questions Source of funding: Not reported Conflict of interest: Not reported	High risk	Knowledge gain	Pre-intervention Mean ± SD: NR Post-intervention Mean ± SD: IG:42.4±4.6, CG:35.9±3.9 Absolute change from the baseline: NR Post-intervention Ratio of the mean (IG/CG): 1.2 P value: 0.001<br><br>Conclusion: OCDE group had better outcome
Hsieh 2006 [45] USA	Setting: University Hospital Medical discipline: Dentistry Participants: 174 Practicing doctors	Study design: Parallel RCT IG: Received pretest, then interactive multimedia computer-based tutorial tailored to dental professionals on DV and associated oral trauma, then posttest. CG: Received pretest, posttest then completed the computer-based tutorial. Instruments: Invalidated modified version of Jefferson Scale for Physicians (Cognitive). Invalidated domestic violence assessment instrument included 24 questions (Knowledge). Technology type/Duration: Multimedia/ Not reported Source of funding: National Institute of Dental and Craniofacial Research, National Institutes of Health	High risk	Cognitive skill gain	Pre-intervention Mean ± SD: IG: 115.18±12.29, CG: 115.83±11.49 Post-intervention Mean ± SD: NR Absolute change from the baseline: NR Post-intervention Ratio of the mean (IG/CG): NR P value: > 0.05<br><br>Conclusion: No difference in the outcome between the two groups

		Conflict of interest: Not reported			
Jensen 2009 [59] Denmark	Setting: University Hospital<br>Medical discipline: ALS course (cardio-pulmonary resuscitation)<br>Participants: 103 Postgraduate Residents	Study design: Parallel RCT<br>IG: Received ALS course then after 6 months received an digital learning software as a booster for ALS skills and knowledge. The software was a self-directed learning program that included a 10–20 min tutorial on how to use the program and 40 individual patient cases, 5 from each of 8 ALS themes. One case per Month. Total 12 individual cases.<br>CG: Received ALS (ALS) course and had baseline assessment.<br>Technology type/Duration: Software or computer based programs/ 12 months<br>Instruments: Validated MCQs questions<br>Source of funding: Laerdal Norway<br>Conflict of interest: No conflict of interest	High risk	Knowledge gain	Pre-intervention Mean ± SD: IG:81.7±6.0, CG:82.6±5.8<br>Post-intervention Mean ± SD: IG:80.3±6.6, CG:81.8±6.6<br>Absolute change from the baseline: IG: -1.4, CG: -0.8<br>Post-intervention Ratio of the mean (IG/CG): 0.98<br>P value: 0.16<br><br>Conclusion: No difference in the outcome between the two groups
Kay 2001 [39] UK	Setting: University Hospital<br>Medical discipline: Dentistry<br>Participants: 95 Practicing doctors	Study design: Solomon three-group design<br>IG-1: Dentists read the radiographs and made treatment decisions then they participated in the computer-aided learning (CAL) package (educational intervention), then re-read (second reading) the graphs.<br>IG-2: Dentists participate in CAL (intervention) then read the radiographs (one reading only).<br>IG-3: Dentists read radiographs (first) and then re-read them (second), then afterwards utilize the CAL.<br>Technology type/Duration: Software or computer-based programs/ Not reported<br>Instruments: Assessed by calculating sensitivity and	High risk	Cognitive skill gain	Pre-intervention Mean ± SD: NR<br>Post-intervention Mean ± SD: NR<br>Absolute change from the baseline: NR<br>Post-intervention Ratio of the mean (IG/CG): NR<br>P value: NR<br><br>Conclusion: No difference in the outcome between the two groups

		specificity of decision making within and between the study groups.<br>Source of funding: Not reported<br>Conflict of interest: Not reported			
Chao 2010 [42] USA	Setting: University Hospital<br>Medical discipline: Obstetrics and Gynecology<br>Participants: 67 Postgraduate Residents	Study design: Parallel RCT<br>IG: Residents completed a pretest of third- and fourth-degree lacerations. After 1 year they received instructional DVD. Intervention group watched DVD within 7 days<br>CG: Residents went through standard clinical experience and training in obstetrics.<br>Technology type/Duration: Video recording/ 7 days<br>Instruments: Validated 18 MCQs<br>Source of funding: None<br>Conflict of interest: No conflict of interest	Unclear risk	Knowledge gain	Pre-intervention Mean ± SD: IG: 69.57±101, CG: 72.70±62.59<br>Post-intervention Mean ± SD: IG:82.18±69.66, CG:73.64±79.97<br>Absolute change from the baseline: IG:12.61, CG:0.94<br>Post-intervention Ratio of the mean (IG/CG): 1.11<br>P value: <0.001<br>Conclusion: OCDE group had better outcome
Gordon 2011 [56] UK	Setting: Postgraduate education and training center<br>Medical discipline: Pediatrics<br>Participants: 162 Practicing doctors	Study design: Parallel non-blinded RCT<br>IG: Participants were given baseline prescribing assessments, then completed the digital learning course designed for the study on pediatric drug prescribing skills, which took 1–2 h. and given 4 weeks to complete it. Followed by second assessment. A final assessment was sent to all participants 8 weeks later.<br>CG: No intervention. Participants were given baseline, 2nd and final prescribing assessments.<br>Technology type/Duration: Software or computer-based programs / 1-2 hours	High risk	Cognitive skill gain	Pre-intervention Mean ± SD: IG: 66±12.6, CG: 67±11.9<br>Post-intervention Mean ± SD: IG:79±12.1, CG:63±13.5<br>Absolute change from the baseline: IG:13, CG: -4<br>Post-intervention Ratio of the mean (IG/CG): 1.25<br>P value: <0.001

		Instruments: Invalidated 10 structured questions.<br>Source of funding: Not reported<br>Conflict of interest: No conflict of interest			Conclusion: OCDE group had better outcome
Nagile 1993 [52] Canada	Setting: University Hospital<br>Medical discipline: Psychiatry<br>Participants: 64 Postgraduate Residents	Study design: Parallel RCT<br>IG: Used decisional aid (videotape) for mental capacity assessment.<br>CG: Performed mental assessment without decisional aid.<br>Technology type/Duration: Software or computer based programs/ Not reported<br>Instruments: Invalidated thematic analysis to assess skills acquisition (The assessment was based on the proportion of agreement between the expert and the participants on the judgement of mental capacity.)<br>Source of funding: Ontario Ministry of Health Research Personnel Development Program Fellowship and by National Health Research Scholar Awards from Health and Welfare Canada<br>Conflict of interest: Not reported	High risk	Cognitive skill gain	Pre-intervention Mean ± SD: NR<br>Post-intervention Mean ± SD: IG:0.87 ± 0.01, CG:0.86 ± 0.01<br>Absolute change from the baseline: NR<br>Post-intervention Ratio of the mean (IG/CG): 1.01<br>P value: 0.81<br><br>Conclusion: No difference in the outcome between the two groups
Lavigne 2011[38] USA	Setting: Teaching hospital<br>Medical discipline: Pediatrics<br>Participants: 24 Pediatrics practices (clinics) - 270 child with ADHD (Practicing doctors)	Study design: Cluster RCT<br>IG: 12 practices (clinics): 137 child received specialized care. Physicians received 2 hours of didactic training on medication management of ADHD in addition to an ADHD specialist provided 1 hour of training to office staff in the use of software which focuses on ADHD Medication Management Program designed for monitoring and guiding medication titration.<br>CG: 12 practices (clinics): 133 child treatment as usual (no intervention).	High risk	Patient's outcome	Pre-intervention Mean ± SD: NR<br>Post-intervention Mean ± SD: NR<br>Absolute change from the baseline: NR<br>Post-intervention Ratio of the mean (IG/CG): NR<br>P value: > 0.05<br><br>Conclusion: No difference in the outcome

		Technology type/Duration: Software or computer based programs/ 12 months<br>Instrument: validated tool; The ADHD Rating Scales-IV and the SNAP-IV.<br>Source of funding: National Institutes of Health [34].<br>Conflict of interest: No conflict of interest.			between the two groups

**Offline computer-based digital learning vs Text-based learning**					
Ottolini 1998 [46]<br>USA	Setting: University Hospital<br>Medical discipline: Pediatrics<br>Participants: 24 Postgraduate Residents	Study design: Parallel RCT<br>IG: Computer program (CD-ROM simulated calls) to teach residents an approach to management of telephone complaints<br>CG: Text reading on management of telephone complaints.<br>Technology type/Duration: Software or computer-based programs/ 180 min<br>Instruments: Assessment comparison of pre- and post-test for responses to 2	High risk	Cognitive skill gain	Pre-intervention Mean ± SD: IG:70.33±8.33, CG:68.46±6.73<br>Post-intervention Mean ± SD: IG:79.08±8.17, CG:69±13.3<br>Absolute change from the baseline: IG:8.75, CG:0.54<br>Post-intervention Ratio of the mean (IG/CG): 1.14<br>P value: <0.03<br><br>Conclusion: OCDE group had better outcome

		emergency pediatrics calls using validated checklist plus patient perception questionnaire from 7 items on Likert scale.<br>Source of funding: Bayer Institute for Health Care Communication<br>Conflict of interest: Not reported			
Balslev 2005 [58] Denmark	Setting: University Hospital<br>Medical discipline: Pediatrics<br>Participants: 12 Postgraduate Residents	Study design: Parallel RCT<br>IG: Watched a brief (2.5-minute) video recording of the patient on problem-based learning (data exploration, theory building, theory evaluation and meta-reasoning)<br>CG: Read a written description of the video recording without watching the video.<br>Technology type/Duration:	High risk	Cognitive skill gain	Pre-intervention Mean ± SD: IG: 307 ±NR, CG: 354±NR<br>Post-intervention Mean ± SD: IG:446 ±NR, CG:305±NR<br>Absolute change from the baseline: IG:139, CG: -49<br>Post-intervention Ratio of the mean (IG/CG): 1.46<br>P value: Significant for each category separately<br><br>Conclusion: OCDE group had better outcome

		Video recording/ 2.5 min<br>Instruments: Validated tool that used thematic analysis to assess the thinking process.<br>Source of funding: Epilepsy and Education Foundation, Department of Pediatrics, Arhus University Hospital<br>Conflict of interest: No conflict of interest			
Rae 2015 [37]<br>UK	Setting: Teaching hospital<br>Medical discipline: Surgery<br>Participants: 32 Postgraduate Residents	Study design: Four arms RCT<br>IG-1: Interactive Simulation<br>IG-2: Non-interactive Simulation (offline computer-based)<br>IG-3: Video Tutorial (offline computer-based)<br>CG: Control Group (written text)<br>Technology type/Duration:	High risk	Cognitive skill gain	Pre-intervention Mean ± SD: IG-2 (non-interactive simulation): IG:8.43±0.79<br>Post-intervention Mean ± SD: IG-2 (non-interactive simulation): IG:40.57±4.07<br>Absolute change from the baseline: NR (different tests)<br>Post-intervention Ratio of the mean (IG/CG):<br>IG-2: 1.36<br>P value: <0.001<br>Conclusion: OCDE group had better outcome

		Software or computer-based programs AND video recording/ Not reported<br>Instruments: non-validated tool of 12 items MCQs for the pretest (Cognitive skills). 36 items checklist for the posttest<br>Source of funding: Not reported<br>Conflict of interest: No conflict of interest			Pre-intervention Mean ± SD: IG-3 (Video tutorial): IG:6.27±2.15, CG: 6.14±2.19<br>Post-intervention Mean ± SD: IG-3 (Video tutorial): IG:27.20± 6.12, CG:29.87±4.70<br>Absolute change from the baseline: NR (different tests)<br>Post-intervention Ratio of the mean (IG/CG):<br>IG-3: 0.91,<br>P value: 0.396<br><br>Conclusion: No difference between in the outcome between the two groups

Offline computer-based digital learning vs Another method of offline digital learning

Bonevski 1999 [57] Australia	Setting: Primary health care centers<br>Medical discipline: General Practice /Family Medicine<br>Participants: 19 Practicing doctors	Study design: Parallel RCT<br>IG: Received computer-based CME on six preventive topics (i.e. screening behaviors and for identifying risk behaviors) and follow-up with an additional feedback on further information on global estimates on the six specific conditions over a period of 2 to 6 weeks.<br>CG: Received the computer-based CME but did not receive any performance feedback until after the completion of the trial.<br>Technology type/Duration: Software or computer-based programs/ 2 to 6 weeks<br>Instruments: Reliable self-reported patient health survey and validated practitioner checklist.<br>Source of funding: The e Commonwealth General Practice Evaluation Program and the NSW Cancer Council.<br>Conflict of interest: Not reported	High risk	Patient's outcome	Pre-intervention Mean ± SD: NR<br>Post-intervention Mean ± SD: Outcomes could not be merged<br>Absolute change from the baseline: NR<br>Post-intervention Ratio of the mean (IG/CG): NR<br>P value: NR<br><br>Conclusion: OCDE group had better outcome

Davids 2014 [65] South Africa	Setting: University Medical discipline: Internal medicine and anesthesiology Participants: 90 Postgraduate Residents	Study design: Parallel RCT IG: Exposed to the revised version of an application for diagnosis and treatment of electrolyte and acid-base disorders. The application consists of case-based tutorials, each consisting of a series of slides, with the navigation controlled by the user. CG: Exposed to the original version of the application. Technology type/Duration: Software or computer-based programs/ 1-38 min Instruments: Invalidated eight open end questions questionnaire (knowledge). Invalidated thematic analysis: task completion rate (cognitive). Source of funding: Supported by a Doctoral Fellowship Award from Stellenbosch University's Faculty of Medicine and Health Sciences. Conflict of interest: No conflict of interest	Low risk	Knowledge gain	Pre-intervention Mean ± SD: NR Post-intervention Mean ± SD: IG:6.9 ±2.5, CG: 7.0±3.1 Absolute change from the baseline: NR Post-intervention Ratio of the mean (IG/CG): 0.98 P value: 0.91 Conclusion: No difference in the outcome between the two groups
				Cognitive skill gain	Pre-intervention Mean ± SD: NR Post-intervention Mean ± SD: IG:12.98±2.97, CG: 13.18±3.6 Absolute change from the baseline: NR Post-intervention Ratio of the mean (IG/CG): 0.98 P value: 0.77 Conclusion: No difference in the outcome between the two groups

Footnote: ACLS : Advanced Cardiac Life support, ADHD: Attention Deficit Hyperactivity Disorder, ALS: Advanced Life Support , ANTS : Anaesthetists' Non-Technical Skills , CG: Control group, CE: Cost-effectiveness, CI: Confidence Interval, , IG: Intervention group, IUD: intrauterine device, MCQs: Multiple choice questions , NA: Not applicable, NR: Not reported, OSCE: The objective structured clinical examination, RCT: Randomized Control Trial , RR: Relative Risk, SD: Standard deviation

References:

48. Boet S. Self debriefing versus instructor debriefing: A prospective randomized trial. Canadian Journal of Anesthesia. 2010;57:S26

43. D'Alessandro M, Galvin JR, Erkonen WE, Albanese MA, Michaelsen VE, Huntley JS, et al. The instructional effectiveness of a radiology multimedia textbook (HyperLung) versus a standard lecture. Invest Radiol. 1993;28:643-8.

55. Davis J, Chryssafidou E, Zamora J, Davies D, Khan K, Coomarasamy A. Computer-based teaching is as good as face to face lecture-based teaching of evidence based medicine: a randomised controlled trial. BMC Med Educ. 2007;20:7-23.

61. Esfahani MN, Behzadipour M, Nadoushan A J, Shariat S V. A pilot randomized controlled trial of the effectiveness of inclusion of a distant learning component into empathy training. Med J Islam Repub Iran. 2014;28:1-6.

62. Farahmand, S Jalili, E Arbab, M Sedaghat, M Shirazi, M Keshmiri, et al. Distance Learning Can Be as Effective as Traditional Learning for Medical Students in the Initial Assessment of Trauma Patients. Acta Med Iran. 2016;54:600-4.

63. Farokhi, M R Zarifsanaiey, N Haghighi, F Mehrabi. E-learning or in-person approaches in continuous medical education: A comparative study. IIOAB Journal. 2016;7:472-6.

51. Isaranuwatchai, W Alam, F Hoch, J Boet. A cost-effectiveness analysis of self-debriefing versus instructor debriefing for simulated crises in perioperative medicine in Canada. Journal of Educational Evaluation for Health Professions. 2017;13:44-.

41. Farrar Michelle, Connolly Anne M, Lawson John, Burgess Annette, Lonergan Amy, Bye Ann M E. Teaching doctors how to diagnose paroxysmal events: a comparison of two educational methods. Med Educ. 2008;42:909-14.

49. Garcia-Rodriguez JA, Donnon T. Using Comprehensive Video-Module Instruction as an Alternative Approach for Teaching IUD Insertion. Fam Med. 2016;48:15-20.

60. Pelayo-Alvarez M, Albert-Ros X, Gil-Latorre F, Gutierrez-Sigler D. Feasibility analysis of a personalized training plan for learning research methodology. Med Educ. 2000;34:139-45.

54. Welke TM, LeBlanc VR, Savoldelli GL, Joo HS, Chandra DB, Crabtree NA, et al. Personalized oral debriefing versus standardized multimedia instruction after patient crisis simulation. Aesthesia & Analgesia. 2009;109:183-9.

47. Platz E, Liteplo A, Hurwitz S, Hwang J. Are live instructors replaceable? Computer vs. classroom lectures for EFAST training. The Journal of Emergency Medicine. 2011;40:534-8.

50. Hards Andrea, Davies Sharon, Salman Aliya, Erik-Soussi Magda, Balki Mrinalini. Management of simulated maternal cardiac arrest by residents: didactic teaching versus electronic learning. Can J Anaesth. 2012;59:852-60.

64. Khoshbaten M, Soleimanpour H, Ala A, Shams Vahdati S, Ebrahimian K, Safari S. Which Form of Medical Training is the Best in Improving Interns' knowledge Related to

Advanced Cardiac Life Support Drugs Pharmacology? An Educational Analytical Intervention Study Between Electronic Learning and Lecture-Based Education. Anesthesiology and pain medicine. 2014;4:e15546-e.

53. Tulsky JA, Arnold RM, Alexander SC, Olsen MK, Jeffreys AS, Rodriguez KL, et al. Enhancing communication between oncologists and patients with a computer-based training program: a randomized trial. Annals of internal medicine. 2011;155:593-601.

40. Millard FB, Thistlethwaite J, Spagnolo C, Kennedy RL, Baune BT. Dementia diagnosis: A pilot randomised controlled trial of education and IT audit to assess change in GP dementia documentation. Australian Journal of Primary Health. 2008;14:141-9.

44. Garrett T J, Selnow G, Dobkin J F, Healton C. Computer-assisted instruction in aids infection control for physicians. Teach Learn Med. 1990;2:2015-218.

45. Hsieh Nancy Kwon, Herzig Karen, Gansky Stuart A, Danley Dale, Gerbert Barbara. Changing dentists' knowledge, attitudes and behavior regarding domestic violence through an interactive multimedia tutorial. Journal of the American Dental Association (1939). 2006;137:596-603.

59. Jensen Morten Lind, Mondrup Frederik, Lippert Freddy, Ringsted Charlotte. Using e-learning for maintenance of ALS competence. Resuscitation. 2009;80:903-8.

39. Kay E J, Silkstone B, Worthington H V. Evaluation of computer aided learning in developing clinical decision-making skills. Br Dent J. 2001;190:554-7.

42. Chao T, Wendel GDJ, McIntire DD, Corton MM. Effectiveness of an instructional DVD on third and fourth-degree laceration repair for obstetrics and gynecology postgraduate trainees. Int J Gynaecol Obstet. 2010;109:9-16.

56. Gordon Morris, Chandratilake Madawa, Baker Paul. Improved junior paediatric prescribing skills after a short e-learning intervention: a randomised controlled trial. Arch Dis Child. 2011;96:1191-4.

52. Nagile G, Silberfeld M, O'Rourke K, Fried B, Corber W, Bombardier C, et al. A Randomized Trial of a Decisional Aid for Metal Capacity Assessments. J Clin Epidemiol. 1993;46:221-30.

38. Lavigne John V, Dulcan Mina K, LeBailly Susan A, Binns Helen J, Cummins Thomas K, Jha Poonam. Computer-assisted management of attention-deficit/hyperactivity disorder. Pediatrics. 2011;128:e46-e53.

46. Ottolini MC, Greenberg L. Development and evaluation of a CD-ROM computer program to teach residents telephone management. Pediatrics. 1998;101:1-6.

58. Balslev T, De Grave WS, Muijtjens AM, Scherpbier AJ. Comparison of text and video cases in a postgraduate problem-based learning format. Med Educ. 2005;39:1086-92.

37. Rae AO, Khatib M, Sarker S, Bello F. The Effect of a Computer Based Open Surgery Simulation of an Inguinal Hernia Repair on the Results of Cognitive Task Analysis Performance of Surgical Trainees: An Educational Trial. Br J Surg. 2015;102 (S1):40.

57. Bonevski B, Sanson-Fisher RW, Campbell E, Carruthers A, Reid AL, Ireland M. Randomized controlled trial of a computer strategy to increase general practitioner preventive care. Prev Med. 1999;29:478-86.

65. Davids MR, Chikte UM, Halperin ML. Effect of improving the usability of an e-learning resource: a randomized trial. Adv Physiol Educ. 2014;38
