# Supplementary material for: Medical Doctors’ Offline Computer-Assisted Digital Education: Systematic Review by the Digital Health Education Collaboration
Source: J Med Internet Res. 2019 Mar 1;21(3):e12998. doi: 10.2196/12998 (PMC6418481; doi:10.2196/12998)
Supplement: Multimedia Appendix 4 [file jmir_v21i2e12998_app4.pdf]

Medical doctors' offline-computer-assessed digital education: a systematic review by the Digital Health Education collaboration

Online Supplementary Document 3

Risk of bias in included studies

Overall, 24 of the 29 included trials (83%) were judged to be at a high risk of bias (Balslev 2005; Boet 2010; Bonevski 1999; D'Allessandro 1993; Esfahani 2014; Farahmand 2016; Farokhi 2016; Farrar 2008; Garrett 1990; Gordon 2011; Hards 2012; Hsieh 2006; Isaranuwatchai 2016; Jensen 2009; Kay 2001; Khoshbaten 2014; Lavigne 2011; Millard 2008; Nagile 1993; Ottolini 1998; Pelayo 2000; Platz 2011;Rae 2015; Tulsky 2011). Two trials were judged to be of unclear risk of bias (Chao 2010; Garcia-Rodriguez 2016), while only three trials were judged to be at low risk of bias (Davids 2014; Davis 2007; Welke 2009).

Allocation (selection bias)

Random sequence generation (selection bias)

Random sequence generation was judged to to be of a low risk of bias in 14 of the 29 studies (48 %) ; nine used computer software to generate the random number sequence (Chao 2010; Davids 2014; Davis 2007; Garcia-Rodriguez 2016; Gordon 2011; Hards 2012; Hsieh 2006; Nagile 1993; Welke 2009) and the remaining five used coin tossing technique (Pelayo 2000), minimization technique (Tulsky 2011), Website random sequence generator (www.randum.org) (Jensen 2009), block randomisation (Rae 2015) or by writing the participants' names on balls that were later pulled out from a bag the inside of which could not be seen (Khoshbaten 2014).

Twelve of the 29 included studies (41 %) (Balslev 2005; Boet 2010; Bonevski 1999; D'Allessandro 1993; Esfahani 2014; Farokhi 2016; Garrett 1990; Isaranuwatchai 2016; Kay 2001; Lavigne 2011; Ottolini 1998; Platz 2011) provided little or no information about the random sequence generation and were therefore classified as having an unclear risk of bias. The remaining three studies (Farahmand 2016; Farrar 2008; Millard 2008); had high risk of bias for random sequence generation. In (Farahmand 2016; Farrar 2008) the volunteers were allocated to either intervention or control groups according to the calendar week and month, while in (Millard 2008) the control group was not randomised.

Allocation concealment (selection bias)

There was no information about the allocation concealment methods in 20 out of 29 trials (69%) (Balslev 2005, Boet 2010; Bonevski 1999; Chao 2010; D'Allessandro 1993; Esfahani 2014; Farokhi 2016; Garcia-Rodriguez 2016; Garrett 1990; Hsieh 2006; Isaranuwatchai 2016; Jensen 2009; Kay 2001; Lavigne 2011; Nagile 1993; Ottolini 1998; Pelayo 2000; Platz 2011; Rae 2015; Tulsky 2011) and therefore these studies were classified as having unclear risk of allocation concealment bias. Five studies (17 %) (Davids 2014; Davis 2007; Gordon 2011; Hards 2012; Welke 2009) used sealed and opaque envelopes for concealment and hence were classified as low risk for allocation concealment bias. The remaining four studies had high risk of selection bias as the allocation concealment was not observed. In two of these four studies (Farahmand 2016; Farrar 2008) the volunteers were arbitrarily allocated to either modality according to calendar week and month, in the second (Khoshbaten 2014) the participants' names were pulled directly from the bag and in the third (Millard 2008) the participants in the control group were not randomised.

Blinding (performance bias and detection bias)

Performance Bias

The risk of bias assessment for blinding of participants and personnel was not assessed because of the nature of the interventions which made blinding of participants and personnel not possible. Furthermore, subjective outcomes such as attitudes, and doctors' satisfaction were not included in the risk of bias assessment for blinding of participants and personnel because the participants' responses are easily affected by person's opinion or concerns of consequences of responding negatively to a program developed by a colleague.

Detection bias

Eighteen out of the 29 RCTs (62%) (Boet 2010; Chao 2010; Davids 2014; Davis 2007; Esfahani 2014; Farahmand 2016; Farokhi 2016; Farrar 2008; Garcia-Rodriguez 2016; Gordon 2011; Hards 2012; Isaranuwatchai 2016; Jensen 2009; Millard 2008; Nagile 1993; Ottolini 1998; Tulsky 2011; Welke 2009) were considered to be at low risk of detection bias. The risk of bias was not only considered low risk in studies where all outcome assessors were blinded but also in studies with unblinded assessors if the method of outcome assessment included no element of interpretation and the classification of the result could be done unambiguously e.g. assessment was by multiple choice test.

Two studies were classified as high risk for detection bias because the assessors were not blinded. On one study (Balslev 2005) the outcome assessment depended on evaluating the thinking processes of the participants which was taped recorded and in the other study (Pelayo 2000) the assessment was conducted individually for each participant and corrected by the tutor in the presence of the participant him or herself, with comments on the answers.The remaining nine studies (31%); (Bonevski 1999; D'Allessandro 1993; Garrett 1990; Hsieh 2006; Kay 2001; Khoshbaten 2014; Lavigne 2011;Platz 2011 Rae 2015) were rated as having an unclear risk of bias due to lack of information about the blinding of the outcome assessors.

Incomplete outcome data (attrition bias)

Because none of the participants were blinded to the intervention, there was high risk of attrition bias for any outcome that relied on active participation and follow-up (e.g. answering a questionnaire on attitudes and satisfaction and taking a knowledge test). However, 22 out of the 29 included studies (76%), were classified as low risk of bias for incomplete outcome data (Balslev 2005; Boet 2010; Chao 2010; D'Allessandro 1993; Davids 2014; Davis 2007;Farahmand 2016;Farokhi 2016; Farrar 2008; Garcia-Rodriguez 2016; Garrett 1990; Hards 2012; Hsieh 2006;Isaranuwatchai 2016; Kay 2001; Khoshbaten 2014; Nagile 1993; Ottolini 1998; Platz 2011; Rae 2015; Tulsky 2011; Welke 2009).These studies either showed no attrition, or reported attrition and exclusion with information provided regarding the reason for not analysing all participants being either similar for the two groups, and/or showed only a small and statistically insignificant difference between the attrition in the two groups. Four studies (15%) were classified as high risk of bias due to the differential drop-out and attrition rates (Bonevski 1999; Gordon 2011; Jensen 2009; Millard 2008). The remaining three studies (12 %) were classified as unclear risk of bias for incomplete outcome data. In (Esfahani 2014 and Lavigne 2011), there was no information about attrition and in third one (Pelayo 2000) the results were expressed as median and the number of participants was not mentioned in the outcome results

Bias in cRCT:

We judged the risk of recruitment bias, possible baseline imbalance and possible loss of cluster as unclear in the cRCT (Lavigne 2011) because no information was provided in the report. Comparability of the CRCT to the RCTs could not be made due to the heterogeneity in the participants and the interventions, in addition to not accounting for clusters in the analysis. We assessed the cRCT to be at high risk of incorrect analysis because the authors did not account for the cluster in their analysis.

Selective reporting (reporting bias)

All of the studies (100%) were rated as low risk of selective reporting bias as the authors reported the results for all the outcomes that were stated in the methods sections. We did not assess publication bias using a funnel plot regression because we did not pool data in meta-analysis.

Other potential sources of bias

Twelve out of the 29 studies (41%) were classified as at high risk of other biases due to one or more reasons. Of these; four studies suffered from the use of invalidated assessment tool which mean there was no objective validation of the outcome results (Farahmand 2016; Hards 2012; Platz 2011; Tulsky 2011), and three studies suffered from unbalanced baseline characteristics which may have affected the outcome due to the small sample size (Nagile 1993; Ottolini 1998; Rae 2015). while the same two studies (Ottolini 1998; Rae 2015) in addition to (Balslev 2005) were classified as at high risk of other biases due to the significant difference in the baseline (pre-test) score between the intervention and control groups. One study suffered from contamination (Pelayo 2000) and in one study the participants were recruited from a convenient sample and were given reimbursement (Hsieh 2006).The study by Khoshbaten 2014 was classified at high risk because the authors used identical pre- and post-tests that may have introduced bias by recall of the answers. While in Millard 2008 two participants changed their practice during the study period resulting in different population audit pre- and post-trial and only three arms of the trial were completed due to small number of participants. Inappropriate administration of an intervention was not assessed in any of the included studies due to insufficient information in the trial report. Seventeen out of the 29 studies (59%) were classified as at unclear risk of other types of bias. Three studies (Farokhi 2016; Kay 2001; Lavigne 2011) suffered from unavailable baseline characteristics for participants to exclude significant differences between intervention and control groups. One study was classified as unclear risk of bias due to possible contamination (Chao 2010). And another study suffered from insignificant unbalanced in the pre-test score between the intervention and control groups (Esfahani 2014) while (D'Allessandro 1993) was classified as unclear risk because the authors used assessment tool with low reliability and It was not clear whether there were more residents or staff in department besides the 49 included in study with the possibility of selection bias.

References

1. Balslev T, de Grave WS, Muijtjens AM, Scherpbier AJ..
   Comparison of text and video cases in a postgraduate problem-based learning format. Med Educ 2005 Nov;39(11):1086-92.
2. Boet S. Self-debriefing versus instructor debriefing: A prospective randomized trial. Canadian Journal of Anesthesia 2010;57:S26.
3. Bonevski B, Sanson-Fisher RW, Campbell E, Carruthers A, Reid AL, Ireland M. Randomized controlled trial of computer strategy to increase general practitioner prevent ive care. Prev Med 1999 Dec;29 (6 Pt 1):478-86.
4. Chao TT, Wendel GD Jr, McIntire DD, Corton MM.. Effectiveness of an instructional DVD on third- and fourth-degree laceration repair for obstetrics and gynecology postgraduate trainees.. Int J Gynaecol Obstet 2010 April;109(1):9-16.
5. D'Alessandro MP, Galvin JR, Erkonen WE, Albanese MA, Michaelsen VE, Huntley JS, McBurney RM, Easley G. The instructional effectiveness of a radiology multimedia textbook (HyperLung) versus a standard lecture. Invest Radiol 1993 Jul;28(7):643-8.
6. Davids MR, Chikte UM, Halperin ML. Effect of improving the usability of an e-learning resource: a randomized trial. Adv Physiol Educ 2014 Jun;38(2):155-60.
7. Davis J, Chryssafidou E, Zamora J, Davies D, Khan K, Coomarasamy A.. Computer-based teaching is as good as face to face lecture-based teaching of evidence based medicine: a randomised controlled trial. BMC Med Educ 2007 Jul;20:7-23.
8. Esfahani M.N, Behzadipour M., Nadoushan A. J., Shariat S. V. A pilot randomized controlled trial of the effectiveness of inclusion of a distant learning component into empathy training. Medical Journal of the Islamic Republic of Iran 14 July 2014;28(65):1-6.
9. Farahmand, S Jalili, E Arbab, M Sedaghat, M Shirazi, M Keshmiri, F Azizpour, A Valadkhani, S Bagheri-Hariri, S. Distance Learning Can Be as Effective as Traditional Learning for Medical Students in the Initial Assessment of Trauma Patients. Acta Medica Iranica 2016;54(9):600-604. [PubMed: 27832693]

10. Farokhi, M R Zarifsanaiey, N Haghighi, F Mehrabi, M. E-learning or in-person approaches in continuous medical education: A comparative study. IIOAB Journal 2016;7:472-476.

11. Farrar Michelle, Connolly Anne M, Lawson John, Burgess Annette, Lonergan Amy, Bye Ann M E. Teaching doctors how to diagnose paroxysmal events: a comparison of two educational methods. Medical education 2008;42(9):909-14.

12. Garcia-Rodriguez JA, Donnon T. Using Comprehensive Video-Module Instruction as an Alternative Approach for Teaching IUD Insertion. Fam Med 2016;48(1):15-20.

13. Garrett T J, Selnow G, Dobkin J. F, Healton C. Computer-assisted instruction in aids infection control for physicians. Teaching and Learning in Medicine 1990;2(4):2015-218.

14. Gordon Morris, Chandratilake Madawa, Baker Paul. Improved junior paediatric prescribing skills after a short e-learning intervention: a randomised controlled trial. Archives of disease in childhood 2011;96(12):1191-4.

15. Hards Andrea, Davies Sharon, Salman Aliya, Erik-Soussi Magda, Balki Mrinalini. Management of simulated maternal cardiac arrest by residents: didactic teaching versus electronic learning. Canadian journal of anaesthesia 2012;59(9):852-60.

16. Hsieh Nancy Kwon, Herzig Karen, Gansky Stuart A, Danley Dale, Gerbert Barbara. Changing dentists' knowledge, attitudes and behavior regarding domestic violence through an interactive multimedia tutorial. Journal of the American Dental Association (1939) 2006;137(5):596-603.

17. Isaranuwatchai, W Alam, F Hoch, J Boet, S. A cost-effectiveness analysis of self-debriefing versus instructor debriefing for simulated crises in perioperative medicine in Canada. Journal of Educational Evaluation for Health Professions 2017;13:44. [PubMed: 28028288]

18. Jensen Morten Lind, Mondrup Frederik, Lippert Freddy, Ringsted Charlotte. Using e-learning for maintenance of ALS competence. Resuscitation 2009;80(8):903-8.

19. Kay E J, Silkstone B, Worthington H V. Evaluation of computer aided learning in developing clinical decision-making skills. British dental journal 2001;190(10):554-7.

20. Khoshbaten Manouchehr, Soleimanpour Hassan, Ala Alireza, Shams Vahdati Samad, Ebrahimian Kimia, Safari Saeid, et al. Which Form of Medical Training is the Best in Improving Interns' knowledge Related to Advanced Cardiac Life Support Drugs Pharmacology? An Educational Analytical Intervention Study Between Electronic Learning and Lecture-Based Education.. Anesthesiology and pain medicine 2014;4(1):e15546.

21. Lavigne John V, Dulcan Mina K, LeBailly Susan A, Binns Helen J, Cummins Thomas K, Jha Poonam. Computer-assisted management of attention-deficit/hyperactivity disorder. Pediatrics 2011;128(1):e46-53.

22. Millard F. B., Thistlehwaite J., Spagnolo C., Kennedy L. R., Baune B. T.. Dementia Diagnosis: A Pilot Randomised Controlled Trial of Education and IT Audit to Assess Change in GP Dementia Documentation. Australian Journal of Primary Health December 2008;14:141-146.

23. Nagile G., Silberfeld M., O'Rourke K., Fried B., Corber W., Bombardier C., Detsky A.. A Randomized Trial of a Decisional Aid for Metal Capacity Assessments. J Clin Epidemiol 1993;46(3):221-230.

24. Ottolini MC, Greenberg L.. Development and evaluation of a CD-ROM computer program to teach residents telephone management. Pediatrics March 1998;101:1-6.

25. Pelayo-Alvarez M, Albert-Ros X, Gil-Latorre F, Gutierrez-Sigler D. Feasibility analysis of a personalized training plan for learning research methodology. Med Educ Feb 2000;34(2):139-45.

26. Platz E, Liteplo A, Hurwitz S, Hwang J. Are live instructors replaceable? Computer vs. classroom lectures for EFAST training. The Journal of Emergency Medicine 2011;40(5):534-538.

27. Rae A.O, Khatib. M, Sarker S, Bello F. The Effect of a Computer Based Open Surgery Simulation of an Inguinal Hernia Repair on the Results of Cognitive Task Analysis Performance of Surgical Trainees: An Educational Trial. British Journal of Surgery 2015;102:40.

28. Tulsky J. A., Arnold R. M., Alexander S.C., Olsen M. K., Jeffreys A. S., Rodriguez K. L., Skinner C. S., Farrell D., Abernethy A. P., Pollak K. I. Enhancing Communication

Between Oncologists and Patients With aComputer-Based Training Program. Ann Intern Med 2011;155:593-601.

29. Welke TM, LeBlanc VR, Savoldelli GL, Joo HS, Chandra DB, Crabtree NA, Naik VN. Personalized oral debriefing versus standardized multimedia instruction after patient crisis simulation. Aesthesia & Analgesia July 2009;109(1):183-189.
