# Supplementary material for: Medical Doctors’ Offline Computer-Assisted Digital Education: Systematic Review by the Digital Health Education Collaboration
Source: J Med Internet Res. 2019 Mar 1;21(3):e12998. doi: 10.2196/12998 (PMC6418481; doi:10.2196/12998)
Supplement: Multimedia Appendix 5 [file jmir_v21i2e12998_app5.pdf]

Medical doctors' offline-computer-assessed digital education: a systematic review by the Digital Health Education collaboration

Online Supplementary Document 4

Evers checklist for economic evaluation

No	**Item**	Yes	No
1	Is the study population clearly described?	√	
2	Are competing alternatives clearly described?	√	
3	Is a well-defined research question posed in answerable form?	√	
4	Is the economic study design appropriate to the stated objective?	√	
5	Is the chosen time horizon appropriate to include relevant costs and consequences?	√	
6	Is the actual perspective chosen appropriate?	√	
7	Are all important and relevant costs for each alternative identified?		√
8	Are all costs measured appropriately in physical units?	√	
9	Are costs valued appropriately?	√	
10	Are all important and relevant outcomes for each alternative identified?	√	
11	Are all outcomes measured appropriately?	√	
12	Are outcomes valued appropriately?	√	
13	Is an incremental analysis of costs and outcomes of alternatives performed?	√	
14	Are all future costs and outcomes discounted appropriately?		√
15	Are all important variables, whose values are uncertain, appropriately subjected to sensitivity analysis?	√	
16	Do the conclusions follow from the data reported?	√	
17	Does the study discuss the generalizability of the results to other settings and patient/ client groups?		√
18	Does the article indicate that there is no potential conflict of interest of study researcher(s) and funder(s)?	√	
19	Are ethical and distributional issues discussed appropriately?	√	

1. Evers Evers S, Goossens M, de Vet H, van Tulder M, Ament A. Criteria list for assessment of methodological quality of economic evaluations: Consensus on Health Economic Criteria. International Journal of Technology Assessment in Health Care. 2005; 21:240-5.
