# Supplementary material for: Medical Doctors’ Offline Computer-Assisted Digital Education: Systematic Review by the Digital Health Education Collaboration
Source: J Med Internet Res. 2019 Mar 1;21(3):e12998. doi: 10.2196/12998 (PMC6418481; doi:10.2196/12998)
Supplement: Multimedia Appendix 6 [file jmir_v21i2e12998_app6.pdf]

Summary of Findings Table 1: Offline digital learning compared with face-to-face learning for doctors' education.

Outcomes	Results	Participants' studies	Quality of evidence [1]	Comments
Knowledge gain	Results of these studies could not be pooled because of the heterogeneity in participants' interventions and outcomes; 4 RCTs[a] showed offline digital learning to have equal effect to face-to-face learning; 3 RCTs showed better gain of knowledge with offline digital learning; 1 trial showed that face-to-face learning has better outcome than digital learning.	495 (247 in intervention and 248 in control groups); 8 RCTs	Very low[b,c,d]	All trials were judged to be at high risk of bias; Indirectness of evidence judged to have affected the estimate of the effect size because of the prior background knowledge of the participants in 2 studies.
Cognitive skills acquisition	Results of these studies could not be pooled because of the heterogeneity in participants' interventions and outcomes; 4 trials showed that there is an equal effect in cognitive skill acquisition; 2 trials showed better skill acquisition in face-to-face group, and 2 studies showed better skill acquisition in offline digital learning group.	375 participants (188 in the intervention and 187 in the control groups); 8 RCTs	Very low[b,c,d]	All trials were judged to be at high risk of bias. Indirectness of evidence judged to have affected the estimate of the effect size because of the prior background knowledge of the participants in 1 trial.
Patients' outcome	Data could not be pooled because of differences in the participants' interventions and the outcomes; The 2 RCTs showed that offline digital	62 participants (32 in intervention group and 30 in control group); 2 RCTs	Low[b,c]	—

	learning had significantly better patients' outcomes.			

Footnote 1: Patient or population: Interns, residents, and physicians; Settings: University hospitals/center, teaching hospitals/center, conference activity; Intervention: Offline computer-based digital learning; Comparison: Face-to-face learning.

Footnote 2: GRADE Working Group grades of evidence: High quality: We are very confident that the true effect lies close to that of the estimate of the effect; Moderate quality: We are moderately confident in the effect estimate: The true effect is likely to be close to the estimate of the effect, but there is a possibility that it is substantially different; Low quality: Our confidence in the effect estimate is limited: The true effect may be substantially different from the estimate of the effect; Very low quality: We have very little confidence in the effect estimate: The true effect is likely to be substantially different from the estimate of effect.

[a]RCT: randomized controlled trial.
[b]Downgraded by (-1) because of high risk of bias.
[c]Downgraded by (-1) because of inconsistency.
[d]Downgraded by (-1) because of indirectness of evidence.

Summary of Findings Table 2: Offline digital learning compared with no intervention for doctors' education.

Outcomes	Results	Participants studies	Quality of evidence [1]	Comments
Knowledge gain	Results of these studies could not be pooled because of the heterogeneity in participants' interventions and outcomes; 3 RCTs[a] showed offline digital learning to be significantly more effective than no intervention in knowledge gain and 1 showed no difference.	401 participants (195 in the intervention group and 206 in the control group); 4 RCTs	low[b,c]	—
Cognitive skills acquisition	Results of these trials could not be pooled because of the heterogeneity in participants' interventions and outcomes; 3 RCTs showed digital learning to have equal effect to no intervention; The fourth RCT showed that digital learning was more effective in cognitive skills acquisition compared with no intervention.	495 participants (258 in the intervention group and and 237 in the control group); 4 RCTs	very low[b,c,d]	All trials are at high risk of bias. Indirectness of evidence judged to have affected the estimate of the effect size because of the previous background knowledge of the

				participants in 2 of the RCTs.
Patients' outcome	It showed that offline digital learning had equal effect to no intervention for patients' outcomes.	Doctors in 24 practices (12 in the intervention group and 12 in the control group); 1 RCT	low[b,e]	The evidence was driven from a single cRCT[f].

Footnote 1: Patient or population: Obstetric residents, medical doctors, and dentists; Settings: University hospitals/center, teaching hospitals/center; Intervention: Offline digital learning; Comparison: No intervention.

Footnote 2: GRADE Working Group grades of evidence: High quality: We are very confident that the true effect lies close to that of the estimate of the effect; Moderate quality: We are moderately confident in the effect estimate: The true effect is likely to be close to the estimate of the effect, but there is a possibility that it is substantially different; Low quality: Our confidence in the effect estimate is limited: The true effect may be substantially different from the estimate of the effect; Very low quality: We have very little confidence in the effect estimate: The true effect is likely to be substantially different from the estimate of effect.

[a]RCT: randomized controlled trial.
[b]Downgraded by (-1) because of high risk of bias.
[c]Downgraded by (-1) because of inconsistency.
[d]Downgraded by (-1) because of indirectness of evidence.
[e]Downgraded by (-1) because of single study.
[f]cRTC: cluster randomized controlled trial.

Summary of Findings Table 3: Offline computer-based digital learning compared with text-based learning for doctors' education.

Outcomes	Results	Participants studies	Quality of evidence [1]	Comments
Cognitive skills acquisition	3 RCTs[a] (4 comparisons) compared OCDE[b] with written text; 1 RCT showed significantly higher cognitive skills posttest scores for participants of offline digital learning compared with text-based learning; Another RCT showed significantly higher cognitive skills posttest scores for text-based learning participants compared with the offline digital	68 participants (43 in the intervention group and 25 in the control group); 3 RCTs	very low[c,d,e]	The evidence was driven from only 1 study with a small number of participants; Indirectness of evidence judged to have affected the estimate of the effect size

	learning; 2 RCTs showed no significant difference in posttest scores between the 2 groups.			because of the prior background knowledge of the participants.

Footnote 1: Patient or population: Residents of pediatrics; Settings: University hospitals/center, teaching hospitals/center; Intervention: Offline computer-based digital learning; Comparison: Text-based learning.

Footnote 2: GRADE Working Group grades of evidence: High quality: We are very confident that the true effect lies close to that of the estimate of the effect; Moderate quality: We are moderately confident in the effect estimate: The true effect is likely to be close to the estimate of the effect, but there is a possibility that it is substantially different; Low quality: Our confidence in the effect estimate is limited: The true effect may be substantially different from the estimate of the effect; Very low quality: We have very little confidence in the effect estimate: The true effect is likely to be substantially different from the estimate of effect.

[a]RCT: randomized controlled trial.

[b]OCDE: offline computer-based digital learning.

[c]One study was downgraded by (-1) because of high risk of bias.

[d]Downgraded by (-1) because of indirectness of evidence.

[e]Downgraded by (-1) because of small number of participants.
